# Caribbean-Wide, Long-Term Study of Seagrass Beds Reveals Local Variations, Shifts in Community Structure and Occasional Collapse

**DOI:** 10.1371/journal.pone.0090600

**Published:** 2014-03-03

**Authors:** Brigitta I. van Tussenbroek, Jorge Cortés, Rachel Collin, Ana C. Fonseca, Peter M. H. Gayle, Hector M. Guzmán, Gabriel E. Jácome, Rahanna Juman, Karen H. Koltes, Hazel A. Oxenford, Alberto Rodríguez-Ramirez, Jimena Samper-Villarreal, Struan R. Smith, John J. Tschirky, Ernesto Weil

**Affiliations:** 1 Instituto de Ciencias del Mar y Limnología, Universidad Nacional Autónoma de México, Cancún, Mexico; 2 Centro de Investigación en Ciencias del Mar y Limnología (CIMAR), Universidad de Costa Rica, San Pedro, Costa Rica; 3 Smithsonian Tropical Research Institute, Panama, Republic of Panama; 4 Discovery Bay Marine Laboratory, Discovery Bay, Jamaica; 5 Institute of Marine Affairs, Trinidad, Trinidad and Tobago; 6 Office of Insular Affairs, Department of the Interior, Washington DC, United States of America; 7 CERMES, University of the West Indies, Barbados, West Indies; 8 Instituto de Investigaciones Marinas y Costeras (INVEMAR), Santa Marta, Colombia; 9 Bermuda Biological Station for Research, St. George, Bermuda; 10 Garrett Park, Maryland, United States of America; 11 Department of Marine Sciences, University of Puerto Rico, Mayaguez, Puerto Rico, United States of America; University of Waikato (National Institute of Water and Atmospheric Research), New Zealand

## Abstract

The CARICOMP monitoring network gathered standardized data from 52 seagrass sampling stations at 22 sites (mostly *Thalassia testudinum*-dominated beds in reef systems) across the Wider Caribbean twice a year over the period 1993 to 2007 (and in some cases up to 2012). Wide variations in community total biomass (285 to >2000 g dry m^−2^) and annual foliar productivity of the dominant seagrass *T. testudinum* (<200 and >2000 g dry m^−2^) were found among sites. Solar-cycle related intra-annual variations in *T. testudinum* leaf productivity were detected at latitudes > 16°N. Hurricanes had little to no long-term effects on these well-developed seagrass communities, except for 1 station, where the vegetation was lost by burial below ∼1 m sand. At two sites (5 stations), the seagrass beds collapsed due to excessive grazing by turtles or sea-urchins (the latter in combination with human impact and storms). The low-cost methods of this regional-scale monitoring program were sufficient to detect long-term shifts in the communities, and fifteen (43%) out of 35 long-term monitoring stations (at 17 sites) showed trends in seagrass communities consistent with expected changes under environmental deterioration.

## Introduction

Seagrass beds are among the most extensive shallow marine coastal habitats worldwide [1]. Their ecosystem services include sustaining diverse faunal communities [1], supporting fisheries [2], providing coastal protection through stabilization of sediments [3], cycling of nutrients [4] and carbon sequestration [5], [6]. In the Caribbean, seagrasses are associated with marine/brackish protected bays and estuaries or reef systems (reef lagoons between the coastlines and the coral reefs). In reef systems, seagrass communities fulfil the above-mentioned services, and additionally provide important ecological linkages with the adjacent coral reefs and/or mangroves. Seagrass communities support the existence of coral reefs through the export of organic materials [7] and provide grazing grounds and/or nurseries for coral reef fishes and other reef fauna [8]–[10]. In addition, associated calcareous macro-algae and epiphytes (algae and invertebrates) on seagrass leaves are major providers of calcium carbonate sediments [11]–[13].

In places where funding resources are particularly limited such as developing countries in the Caribbean, bio-indicators can provide warning of changes in the biological condition of a coastal system and are thereby valuable to natural resource managers [14]. Despite such relevance, comprehensive spatio-temporal analyses of bio-indicators in tropical countries are scarce because of the lack of long-term monitoring data. Seagrasses are widely distributed, rooted in the substrate and respond to changes in the environment in terms of morphology and population characteristics; thus they can serve as biological indicators (or bio-indicators) for assessing changes in the status of coastal systems [15]. Retreat of seagrass beds to shallower areas, with a consequent reduction in coverage or biomass has been used as an indicator of decreasing water clarity [16], [17]. Seagrasses respond to nutrient enrichment physiologically by increasing N or P content [18], [19], changing morphology such as leaf width [20], [21] and changing biomass distribution between above- and below-ground plant parts [22], [23]. Changes in water quality (clarity, salinity or nutrients) result in changes in species composition [22]–[24] and density of the foliar shoots of the seagrasses [25].

This study presents the results of a long-term (1993–2007, with some continuing to the present) Caribbean-wide seagrass monitoring initiative: the Caribbean Coastal Marine Productivity (CARICOMP) program. The program was established in 1992 to study land-sea interaction processes and to monitor changes through time in the productivity and structure of the three principal tropical coastal ecosystems: mangroves, seagrasses and coral reefs, with the ultimate goal of providing scientific information for management of coastal resources [26]. The CARICOMP program has generated a Caribbean-wide dataset using a simple, low-cost but standardized sampling protocol, consistent among sites over time. While the Caribbean region corresponds to the “Tropical Atlantic” seagrass bioregion [27] which has relatively high species diversity (10 species, [28]), most CARICOMP seagrass study areas were shallow reef lagoons dominated by two species (*Thalassia testudinum* and *Syringodium filiforme,* [29]). The present work aims to document changes within seagrass communities at unprecedented spatial and temporal (more than a decade) scales. We demonstrate that the low-cost standardized CARICOMP sampling protocol (consistent among sites and over time) can provide, in addition to the responses of seagrass communities to season and climate, evidence of deterioration of the environment along Caribbean coastlines.

## Materials and Methods

### Ethics Statement

Sample permits were issued by Florida Keys National Marine Sanctuary for USA-Florida Keys (site 2), SAGARPA for Mexico-Puerto Morelos (site 5, since 2003), Ministerio del Ambiente de Costa Rica for Costa Rica (site 21, since 2000); specific permissions were not required for any other site or date, and the field studies did not involve endangered or protected species.

**Figure 1:**
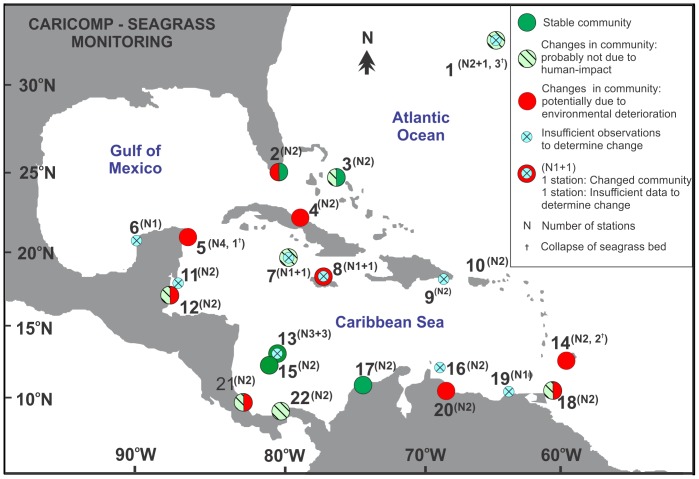
Map of CARICOMP seagrass sites, ordered according to latitude. 1. Bermuda, 2. USA-Long Key, 3. Bahamas-San Salvador, 4. Cuba-Cayo Coco, 5. Mexico-Puerto Morelos, 6. Mexico-Celestun, 7. Cayman Islands-Grand Cayman, 8. Jamaica-Discovery Bay, 9. Dominican Republic-Parque Nacional Este, 10. Puerto Rico-La Parguera, 11. Belize-Turneffe Island, 12. Belize-Twin Cays/Carrie Bow Cay, 13. Colombia-Isla Providencia, 14. Barbados-St. Lawrence, 15. Colombia-Isla San Andres, 16. Curaçao-Spaanse Water, 17. Colombia-Chengue Bay, 18. Tobago-Bon Accord Lagoon, 19. Venezuela-Isla de Margarita, 20. Venezuela-Morrocoy, 21. Costa Rica-Cahuita, 22. Panama-Isla de Colon.

The sampling protocols and organization of the monitoring network are described in CARICOMP [26], [29]. Seagrass monitoring was conducted at 22 sites with 52 sampling stations (Fig 1) from 1993 until 2012, although many concluded the monitoring program before 2007 (Table S1). At each site, generally two *Thalassia testudinum-*dominated stations were selected by the participants; one station representing the most developed *T. testudinum* bed (“high productivity”) and the second an average or typical bed. However, some sites had only one station, whereas others had up to six stations (Table S1). Bi-annual sampling intervals were specified in the protocol, but participation in the program was voluntary, and sampling frequencies and periods varied among sites (Table S1).

Growth and productivity of the seagrass *T. testudinum* were determined in 4–6 haphazardly placed quadrats (10×20 cm) per station. The leaves were marked just above the colorless basal sheath (or at the level of the quadrats) by punching one or two holes with a syringe needle and left to grow for 7–14 days. After this time, the foliar shoots were counted and the leaves were cut at the levels of the previous basal marks. Alternatively, the leaves were marked again at the base, and the entire foliar shoots (including sheaths) were retrieved from the sediments, and the foliar shoots were counted in the laboratory. In the laboratory, leaf tissues were separated into new growth (newly emerged leaves and leaf sections below the mark of old leaves) and old fractions. The epiphytes were removed by rinsing the blades in a 10% acid solution and/or scraping with a razorblade. The leaf fractions were dried and dry weight was determined. The dry weights of new growth fractions represented the production (g m^−2^ d^−1^) and the combined weight of both fractions corresponded with leaf biomass (g m^−2^). Based on expected changes in growth due to the solar cycle, sampling was planned twice a year: once in the high-growth season (March through August) and once during the low-growth season (September through February). Annual productivity rates were determined by averaging the daily productivity rates (per m^−2^) of all samples collected during the low- and high growth seasons for each year and multiplying by 365.

Biomass of the seagrass community was determined by taking two to four core samples at each station with a PVC or steel corer 15–20 cm in diameter (depending on site). The seagrasses (*T. testudinum* and “other grasses”, mostly *Syringodium filiforme*) were separated into above- and below-ground fractions. Above-ground fractions were also separated and analyzed for the rooted calcareous and fleshy algae (below-ground parts were excluded from the analysis). The fractions were cleaned and dried before the weight was determined. The calcareous algae were decalcified in a 10% acid solution before being dried and weighed to determine their somatic weight. Annual biomass (per m^−2^) was determined as the means of all samples collected during a year.

Possible spatial patterns for mean daily productivity rates of *T. testudinum* related to latitude or Physical Environments of the Caribbean Sea (PECS) defined by Chollet et al. [30] were explored using a Random Forest analysis [31]. The standard setting for the analysis defined by R v. 2.15.3 (creation of 500 randomly selected decision trees) and overall mean values (Table S2) for the stations were used for this analysis. The same analyses were applied to total (above-and below-ground) biomass of the seagrass community (data in table S3). Posteriorly, the terms latitude, depth and Secchi reading (table S2) were combined (latitude*depth*Secchi reading) to discern whether a combination of these terms could explain *T. testudinum* productivity or total community biomass.

Intra-annual variation in productivity of *T. testudinum* was determined for twenty-four stations at eleven sites that had ten or more sampling events. General mean productivity was computed for each of the twenty-four stations as the average of all measurements of productivity at that station. Intra-annual variation (ΔP) was determined by calculating the deviation from the general mean productivity for each sampling event (per station), expressed as the percentage of the general mean productivity. ΔP was plotted separately for high- and low- growth season at each station. For each degree of latitude, a One-sample t-test was applied to test whether ΔP differed from zero. At Long Key, Florida (Site 2), and Bon Accord Lagoon, Tobago (Site 18), sampling was conducted more than twice a year. Correlations between mean monthly Sea Surface Temperatures, (SST), hours of daylight and growth rates per shoot (g dry shoot^−1^d^−1^) were determined for each station at these two sites.

An interim report of the CARICOMP program identified increased terrestrial run-off (sewage, fertilizers and/or sediments) as the major and most prevalent anthropogenic influence in the monitoring region [29]. Consequences of increased terrestrial run-off into coastal waters are mainly increasing nutrients loads and/or decreasing water clarity. The following indicators of long-term changes in community and seagrass parameters were used as indicators of potential changes in coastal conditions: 1) Total (above-, and below ground) community biomass (seagrasses and algae), 2) Relative dominance (above-ground biomass seagrass/above-ground community biomass) of faster-growing seagrasses (classified as “other seagrasses” in the CARICOMP protocol, but mainly consisting of *S. filiforme*), 3) Relative dominance of faster growing fleshy algae, 4) Above-ground biomass relative to total biomass of the seagrasses (the most abundant seagrass *T. testudinum* was used for this assessment), 5) Productivity of *T. testudinum* and 6) Foliar shoot density of *T. testudinum*. Responses to changes in the environment depend on local settings [14], but combinations of consistent trends in two or more of the above-mentioned parameters may indicate environmental degradation. Both decrease in water clarity and increase in nutrients were expected to increase the dominance of faster growing seagrasses (parameter 2) or algae (parameter 3), and/or increase the relative investment in above-ground biomass (parameter 4). Total community biomass (parameter 1) and productivity of *T. testudinum* (parameter 5) are expected to decrease at increasing turbidity and to increase at increasing nutrient input into oligotrophic or mesotrophic systems. Foliar shoot density of *T. testudinum* (parameter 6) is expected to decrease with increasing turbidity. Significant slopes of linear regressions for each of these parameters versus year were considered to indicate a consistent pattern of change over the sampling period. These long-term trends were determined only when sampling covered at least five years (although intermittent at some stations) sampling effort with at least six sampling events, and included 35 stations at 17 sites.

## Results

**Figure 2:**
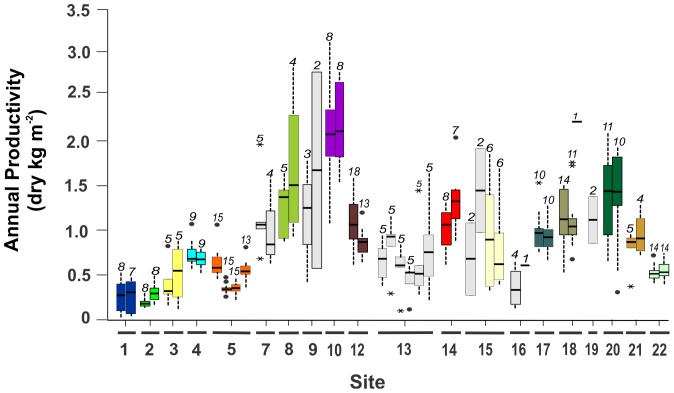
Annual leaf productivity of *Thalassia testudinum* per sampling station. The stations are grouped per site (underlined, 1–6 stations per site), and stations only sampled during one season are excluded. The boxes and vertical bars represent inter-annual variation. The horizontal lines correspond with the median values, 50% of the cases are within the box limits and the vertical bars indicate the smallest or largest values that are not outliers, • represent values more than 1.5 box lengths from lower/upper box limit, and * represent values more than 3 box-lengths from lower/upper box limit. The digits above the bars indicate N (the number of sampling years). Grey bars represent stations that were not included in the long-term analysis.

**Figure 3:**
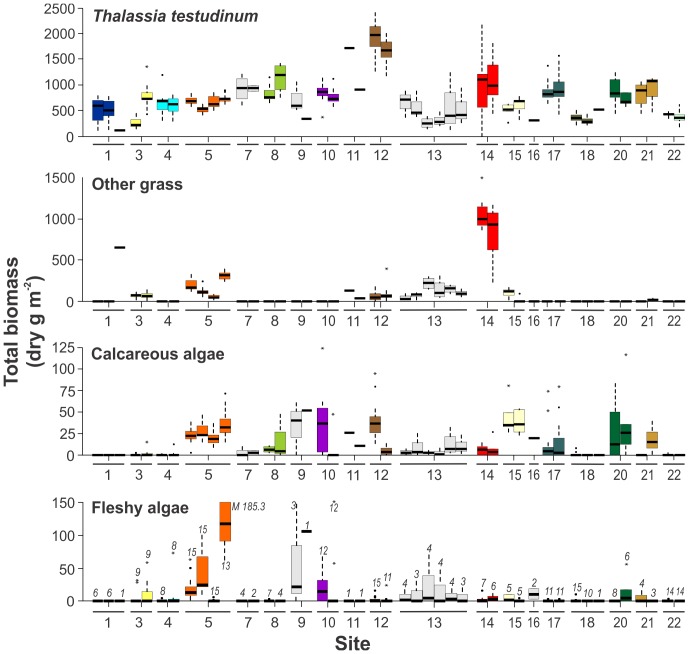
Total (above- and below-ground) biomass of the principal components of the community per sampling station grouped per site. Other grass: species of seagrass other than *Thalassia testudinum*, mostly *Syringodium filiforme*. Somatic (decalcified) above-ground weight of the calcareous algae is considered. The boxes and bars represent inter-annual variation, and stations with only one sampling event are excluded. The digits above the bars in the bottom graph indicate N (the number of sampling years). M median of fleshy algae at site 5-station 13. See legend of Fig. 2 for further explanation.

Annual productivity of leaves of *Thalassia testudinum* varied by an order of magnitude among the stations: lowest productivity was registered at Long Key, Florida Keys, USA (site 2-station 4, < 200 g dry m^−2^ y^−1^) whereas highest leaf productivity (> 2000 g dry m^−2^ y^−1^) was attained at Puerto Rico (site 10-stations 21 and 22) and Tobago (site 18-station 45, Fig. 2, Table S2). Community above-ground biomass varied over 20-fold among stations (Table S3), from 16 g dry m^−2^ at a mono-specific *T. testudinum* bed at Bermuda to 325 g dry m^−2^ at a coastal fringe in Puerto Morelos, Mexico, where 59% of biomass was accounted for by the large fleshy algae *Avrainvillea* spp. (Fig. 3, Table S3). The highest total (above-, and below ground) biomass of *T. testudinum* was registered at Twin Cays, Belize (1960 g dry m^−2^, Fig 3). Inter-annual variations in both productivity and total biomass were considerable at all sampling stations (Figs. 2, 3). Of the 52 stations included in the analysis of biomass, only five (<10%) were monospecific beds of *T. testudinum* (without other seagrasses or macro-algae apart from epiphytes, Fig. 3, Table S3), indicating that the seagrass vegetative communities in the tropical Atlantic reef systems are typically multi-species associations.

No clear classification trees could be constructed relative to latitude for *T. testudinum* leaf productivity and total (above-and below-ground) community (seagrass and macro-algae) biomass. The Random Forests only explained 32.5% (productivity) or 57.9% (biomass) of the variance in the data. When depth and Secchi readings were added as terms, the fits of the models increased for productivity (52.8% of variance explained) and for biomass (59.8% of variance explained). The CARICOMP seagrass sites were in 10 of the 16 physicochemical provinces in the Caribbean defined by Chollett et al. [30], and the models constructed for productivity and PECS did not result in a precise fit either for productivity (34.8% of the variance was explained) or total community biomass (35.1% of the variance).

**Figure 4:**
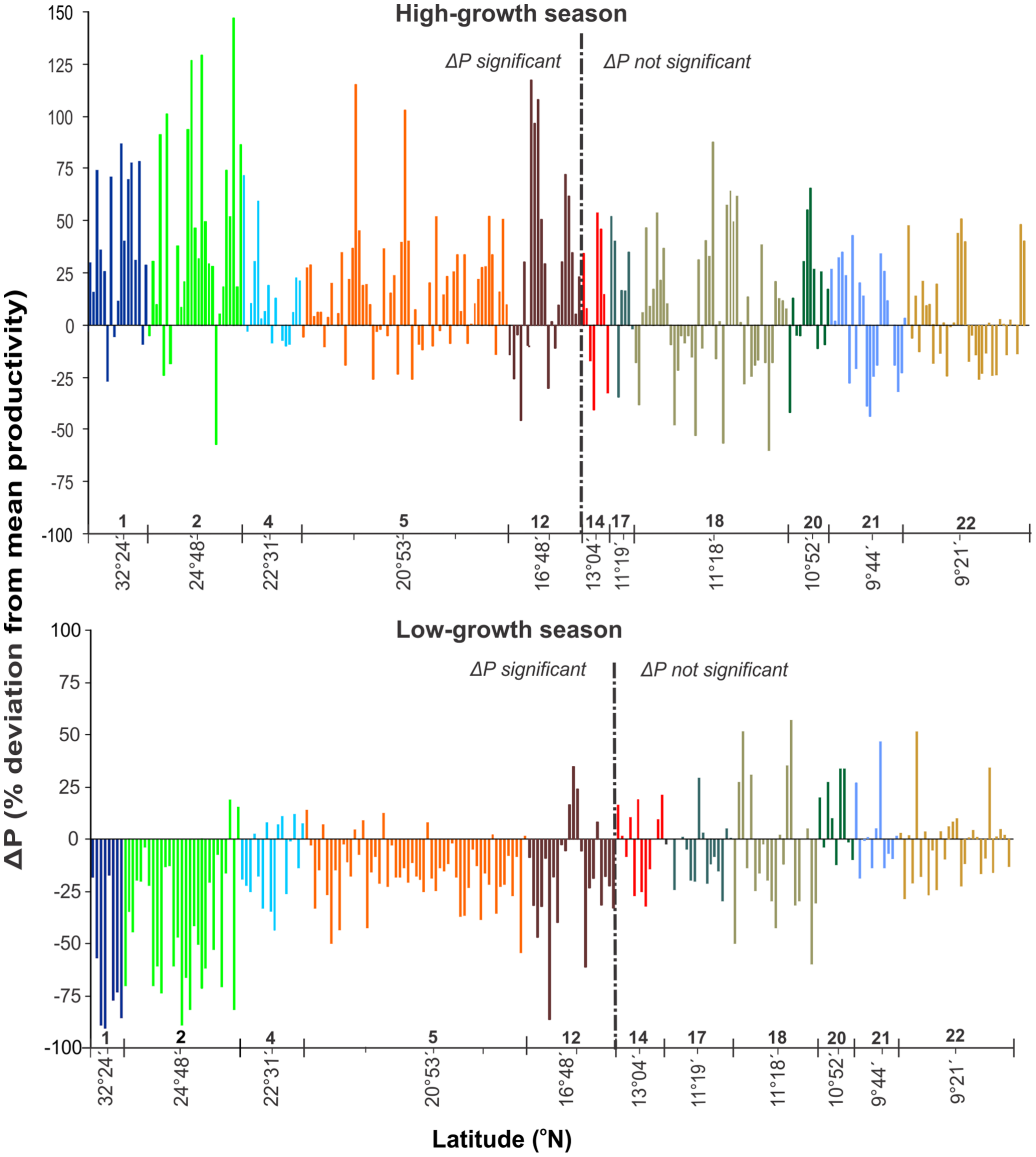
Deviations from general mean leaf productivity (ΔP) of *Thalassia testudinum* per station during High growth season (May-September at site 1 and 2, March –August at all other sites) and Low growth season (October-April at sites 1 and 2, September-February at all other sites). See Table S4 for significance differences of ΔP. Only stations with at least 10 sampling events were included. Numbers above the X-axis indicate site number, and the minor ticks indicate the different sampling stations at those sites.

Intra-annual variation in leaf productivity was evident at latitude 16°48′ (Belize) and higher (Fig. 4, Table S4). At Long Key, Florida (site 2) correlations among mean monthly shoot growth rates of *T. testudinum,* mean monthly SST and median hours of daylight were significant (Figure S1, Table S5). None of these correlations was significant at the more southern Bon Accord Lagoon in Tobago (site 18).

**Figure 5:**
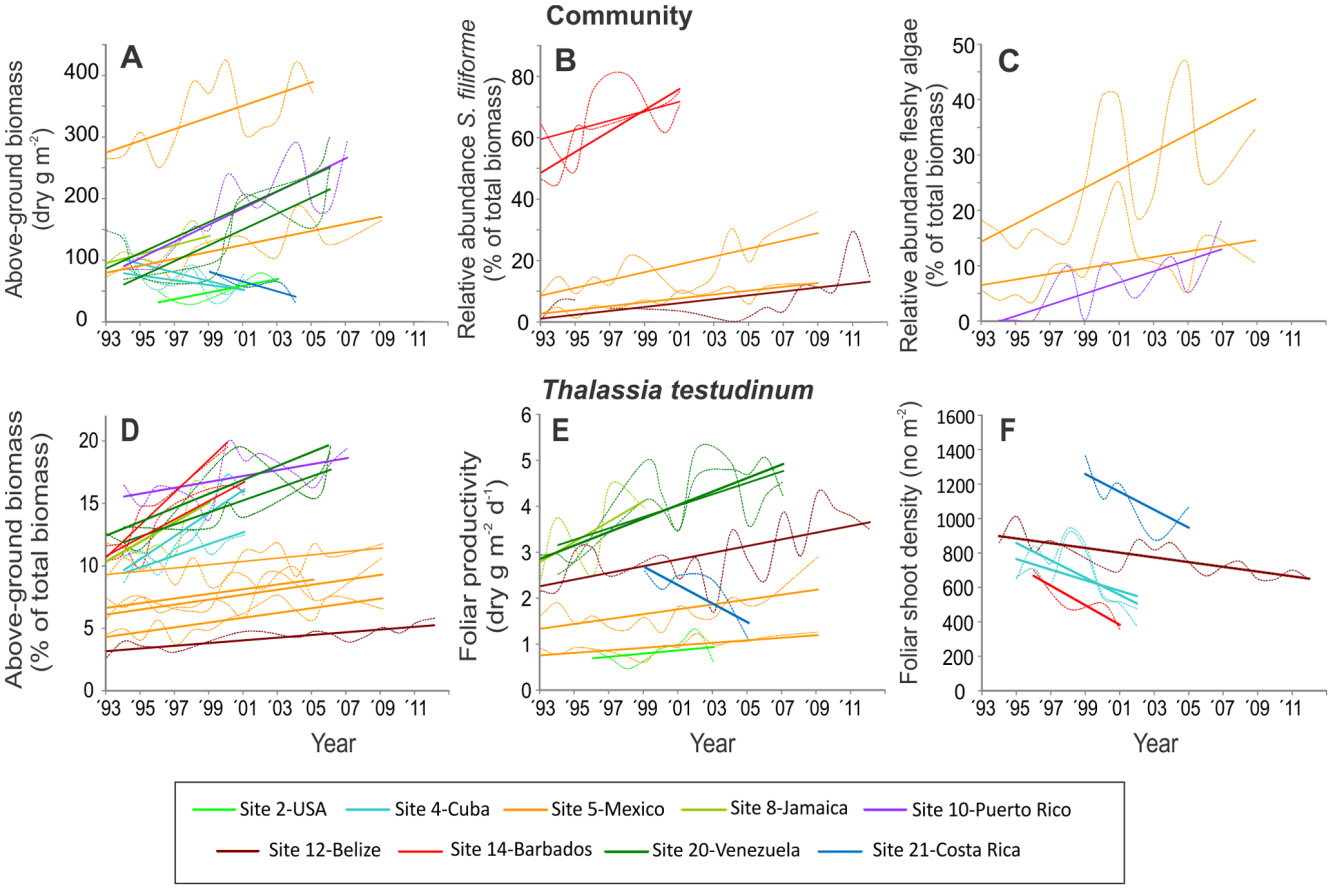
Significant long-term trends in seagrass attributes and community parameters at CARICOMP monitoring stations across the nine sites that showed changes consistent with deterioration of the environmental conditions. The broken smoothed lines connect annual average values and serve to illustrate the inter-annual variability in the data. Data from all samples per year (N = 4-9, Table S3) were used to determine the regression lines (Table S6). D. For Site14, the relationship was determined for the more persistent *Syringodium filiforme*.

**Figure 6:**
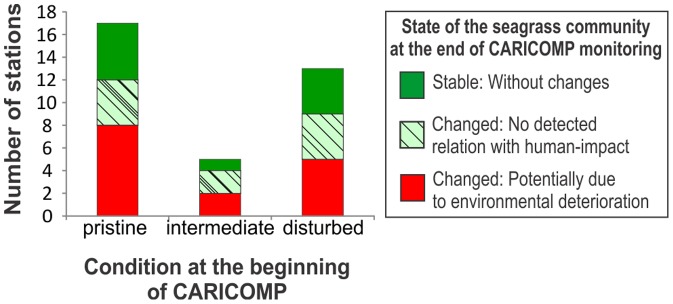
State of the long-term monitoring stations at the beginning (1993) and end (2007–2012) of the CARICOMP program. Pristine: Relatively undisturbed stations at the start of the program. Intermediate: Stations moderately disturbed by human-impact at the beginning of the program. Disturbed: Stations which had undergone chronic human-induced impacts before the initiation of the monitoring program.

The seagrass communities at the majority (25) of the 35 stations included in the analysis for longer-term trends in the community showed changes in at least one of the six selected parameters (Table 1, Table S6). At six stations the seagrass beds collapsed: in Bermuda (3 stations) the decline was due to excessive grazing by sea turtles; in Barbados (2 stations) poor water quality followed by a population explosion of sea urchins and subsequent storms were responsible; and in Mexico, a coastal bed (1 station) was buried by sediments during a hurricane. Most monitoring stations (46 out of 52) were exposed at least once to a major meteorological event (hurricane or tropical storm, Table S1) during the study period, but apart from the above-mentioned collapse of communities in Mexico and Barbados (where the storms were not the main cause of collapse), minor impacts of storms were registered only at Belize-station 26 and Venezuela-stations 47 and 48 (Table 1). At 15 (43%) out of 35 studied stations (Table 1, Fig. 5), changes in the seagrass beds were consistent with hypothesized change scenarios of increased turbidity (Site 4-Stations 8 & 9 and Site 21-Station 49) or increased nutrient input (Site 2-Station 5; Site 5-Stations 10 thru13; Site 8-Station17; Site 10-Station 21; Site 12-Station 25; Site 14-Stations 33 & 34 Site 20-Stations 47 & 48). Most stations that showed shifts in community structure consistent with environmental degradation were reported to have received little or only moderate human-induced impacts at the onset of the study (Fig. 6).

**Table 1:** Long-term trends at CARICOMP seagrass stations, including observations on disturbances.

Site	Station	Country/Territory (sampling period: yr-yr)	Biomass	Rel. ab. - Other seagrass	Rel. Ab. - Fleshy algae	% Above/Total Biomass	Productivity	Shoot density		Condition at beginning of monitoring	
1	1✞✞✞	Bermuda	D	-	-	D	D		I	PRIST	The pristine seagrass beds have showed catastrophic declines since 1997, and they were extirpated in 2001, likely caused by excessive grazing by the green turtle *Chelonia mydas* ^1,2^.
	2✞✞✞	(94-02)	D	-	-	D	D		D		
2	4	USA	-	-	-	-	n		n	INT	Florida Keys suffer from eutrophication through groundwater contamination from septic tanks^3,4^; only the inshore station 5 showed indications of disturbance.
	5^*^	(96-03)	I^N^	-	-	-	I^N^		I		
3	6	Bahamas	n	n	-	n	-		-	EUTR	San Salvador had plantations, but has become a tourist destination since 1970s ^5^.
	7	(/94-06)	n	D	-	n	-		-		
4	8*	Cuba	D^T^	-	-	I^NT^	n		D^T^	PRIST	Cayo Coco was considered as pristine, but increasing sedimentation (decreased light) could have forced changes in seagrass beds^5^.
	9*	(94-02)	D^T^	-	-	I^NT^	n		D^T^		
5	10*	Mexico	n	I^NT^	I^NT^	I^NT^	I^N^		I	PRIST	Increased eutrophication through ground-water discharge likely forced changes in the seagrass beds at Puerto Morelos reef lagoon^6,7,8^. Vegetation at the coastal fringe (station 13) was buried during hurricane Wilma (2005)^7^.
	11*	(93-09)	I^N^	n	I^NT^	I^NT^	n		I		
	12*		n	I^NT^	-	I^NT^	I^N^		n		
	13*✞✞✞		I^N^	n	n	I^NT^	n		n		
7	15	Cayman I.	-	-	-	-	I^N^		D^T^	INT	The high number of visitors to the Grand Cayman compromise carrying capacity and cause environmental degradation, but not in protected CARICOMP areas^9^.
		(97-03)									
8	17*	Jamaica	I^N^	-	-	I^NT^	I^N^		-	DIST	Discovery Bay has been affected by terrestrial runoff from agricultural developments and possibly by proliferation of urban developments without a central sewage system^9^.
		(93-99)									
10	21*	Puerto Rico	I^N^	-	I^NT^	I^NT^	n		n	INT	Residential and tourism developments at and near La Parguera have increased terrigenous sediment load, and raw- and secondary sewage effluents from a treatment plant^9^ possibly changed the seagrass community at station 21.
	22	(94-06)	n	-	n	I^NT^	n		n		
12	25*	Belize	n	I^NT^	n	I^NT^	I^N^		D^T^	PRIST	Both sites have been subjected to loss of water clarity due to increased input of sediments and nutrients from coastal development and agriculture. *T. testudinum* shoot density has declined at Site 25(Twin Cays) possibly due to increased sedimentation^10^. Station 26 (Carrie Bow Cay) was scoured by Hurricane Mitch (1998) but recovering^11^.
	26	(93-12) (97-12)	n	n	n	I^NT^	n		I		
13	29	Isla	n	n	-	n	n		n	PRIST	Isla Providencia (Columbia) was for a long time sparsely inhabited; recently tourism has increased, but the changes in seagrass beds were not consistent with environmental degradation. Hurricane Beta passed close by, but the seagrasses received no impact^12.^
	30	Providencia	n	D	-	n	n		n		
	31	(00-07)	n	n	-	n	D^T^		n		
14	33*✞✞✞	Barbados	n	I^NT^	-	I^NT^	n		D^T^	DIST	St. Lawrence has been affected by anthropogenic activities since 1880s from sugar cane cultivation, residential developments and eutrophication. Combined effects of increased sedimentation due to frequent flushing of a new sewage pipe system, excessive sea urchin grazing and storms caused collapse of the seagrass beds^5, 13^.
	34*✞✞✞	(93-01)	n	I^NT^	-	I^NT^	n		-		
15	37	San Andres	n	n	-	n	-		I	DIST	San Andres Island (Colombia) is the most densely populated oceanic island in the Caribbean. Past population increase with poorly planned development resulted in degradation of coastal ecosystems^5^ The seagrass community did not show changes.
	38	(99-07)	n	n	-	n	-		n		
17	41	Colombia	n	-	-	n	n		n	PRIST	Seagrass beds, mangroves and coral reefs at Chengue Bay are healthy and stable^14^. Abundant *Halimeda opuntia* before 1996 disappeared without obvious cause^5^.
	42	(94-05)	n	-	-	n	n		n		
18	43	Tobago	n	-	-	n	n		n	DIST	Bon Accord Lagoon has received impacts from tourism and sewage for ∼50 y^9^. But apart from a decrease in productivity at station 45, the seagrass conditions were stable.
	44	(92-07)	n	-	-	n	D^T^		n		
20	47*	Venezuela	I^N^	-	-	I^NT^	I^N^		I	DIST	Morrocoy Park has been subjected to increasing land-based constructions, mangrove demolition and sewage effluents since 1970s^9^. Heavy rainfall in 1999 caused loss of seagrass leaves, but recovery followed within months^5^.
	48*	(93-06)	I^N^	-	-	I^NT^	I^N^		I		
21	49*	Costa Rica	D^T^	-	-	n	D^T^		D^T^	PRIST	The Limón earthquake in 1991 affected the seagrass beds at Cahuita which fully recovered after 1 y^5^. Seagrass beds at Station 49 may have deteriorated due to increased turbidity by sewage load, sediments and fertilizers (citrus and banana farming)^15, 16^.
	50	(99-05)	-	-	-	-	D^T^		n		
22	51	Panama	I^N^	-	-	-	n		n	DIST^16^	The narrow *Thalassia testudinum* bed fringing a mangrove swamp showed increased biomass over the years, which may be attributed to natural causes^17^.
	52	(99-06)	I^N^	-	-	-	n		I		

Biomass: total above-ground biomass of the community; Rel abund: relative abundance (biomass) of faster growing seagrass and algal species; Other seagrass: seagrass species other than *Thalassia testudinum* (mostly *Syringodium filiforme*); % Above/Total Biomass: percentage of above-ground of total biomass of *T. testudinum* (*S. filiforme* for site 14, because *T. testudinum* was absent in later years at station 33); Productivity: productivity of leaves of *T. testudinum*. ??? Collapse of seagrass bed, * seagrass beds showed changes that potentially indicate with human-induced environmental deterioration. Trends: **I** increase, **D** decrease, n without change, - not determined, **^N^** expected change due to increasing nutrient load, **^T^** expected change due to increasing turbidity, **^NT^** expected change due to either increasing turbidity or nutrient load, changes without symbol were not consistent with expectations of water quality deterioration (See text for further explanation). Conditions at the beginning of monitoring: PRIST (relatively) pristine (undisturbed by humans); INT Moderate disturbance; DIST Disturbed (eutrophication, terrestrial runoff, or overfishing, from [26], [29], [47]). See Table S6 for information on regression lines. Source: 1. [48], 2. [49], 3. [50], 4. [51], 5. [29], 6. [52], 7. [33], 8. [53], 9. [38], 10. [44], 11. Pers. Obs. K. Koltes, 12.Pers.Obs. H.A Oxenford, 13. [54], 14. [55], 15. [56], 16. [57], 17. [58].

## Discussion

The CARICOMP monitoring program shows wide variation in seagrass productivity and biomass across the Caribbean, reflecting the different environmental settings among the sampling sites, although most were associated with coral reef systems. This study included a broad spectrum of seagrass community types dominated by *Thalassia testudinum*, from highly productive almost mono-specific beds to multi-species communities with several seagrass species and benthic macro-algae. The physicochemical provinces (PECS) defined by Chollett et al. [30] could not reliably predict the mean leaf productivity of *T. testudinum* or total biomass of the community. The 16 PECS were defined based on sea surface temperature, water clarity (from satellite images), salinity, wind-driven exposure and exposure to hurricanes. The criteria for the classification into these 16 provinces (PECS) likely did not include all relevant parameters that determine seagrass development. For example, Zieman et al. [32] suggested that in the Caribbean higher standing crops may be expected at sites with relief and considerable rainfall that supply nutrients for the development of larger plants, such as Jamaica, Puerto Rico, Belize, Venezuela (Morrocoy) and Panama. Latitude determines water temperatures and light regimes, and it was a better predictor for community biomass (but not productivity of *T. testudinum*) than the PECS. Combining latitude with local depth and mean Secchi reading (indicator for water transparency) resulted in more precise predictions; but less than 60% of the variances in mean leaf productivity of *T. testudinum* or total community biomass were explained by these combined predictors, suggesting that other factors also influence the leaf dynamics of *T. testudinum* and status of the seagrass communities.

Intra-annual changes in the growth of *T. testudinum* were registered at latitude 16°48′N (Site 12-Belize) and higher, and they appeared to be mainly driven by the seasonal solar-cycle. Also, at the northerly Florida Keys site, the initiation of the ‘high-growth season’ shows a lag of 1–2 months in comparison with more southerly sites (Figure S1), most likely in response to relatively low temperatures with corresponding reduced growth rates of *T. testudinum* until May (Table S5).

Most seagrass beds in this study have been exposed to hurricanes or major storms during the monitoring period (Table S1, Figure S2), but they were strikingly resilient. The seagrasses at a few stations were negatively affected (see Table 1), but recovery was rapid (within 1 to several years). Eradication only occurred at one station in a narrow (20–50 m wide) coastal fringe in Puerto Morelos, Mexico, during Hurricane Wilma (2005) by burial below ∼1 m of sand [33]. However, recovery of this seagrass bed is now in progress (Van Tussenbroek, unpublished data). The vegetation at most stations consisted of *T. testudinum* dominated beds in sub-tidal reef-systems, where *T. testudinum* is a large and persistent seagrass that invests much of its biomass in below-ground tissue, which aids in firm anchorage of the plants [34] and stabilization of the sediments. Seagrass beds in the tropical or sub-tropical Atlantic that are not associated with reefs are often dominated by less robust seagrass species, such as those in the Gulf of Mexico, may be much more susceptible to damage or destruction by hurricanes or storms [Heck et al., 1996 (in [35]), [36]–[38], although there are exceptions [35].

Forty-three percent of the seagrass communities at the 35 long-term study stations at nine sites show changes that potentially indicate degradation of the environment between 1993 and 2007 (2012 for some stations, Table 1, Fig. 5). These changes over a relatively short time-span (6–18 years) across many sites is a worrying trend, particularly because most of these sites were only moderately disturbed by humans at the outset of the study (Fig. 6). Only two originally undisturbed sites, Colombia-Isla Providencia (site 13, Stations 29–31), and Colombia-Chengue Bay (site 17, stations 41 and 42) remained in ‘pristine’ condition up to the end of the monitoring period (2007 and 2005 respectively, Table 1). Several sites, such as Bahamas-San Salvador (site 3), Colombia-Isla San Andres (site 15), Tobago-Bon Accord lagoon (site 18), Panama-Isla de Colon (site 22), have been impacted by human development for decades or more than a century [39], but we did not detect indications of further degradation during the study period.

The consequences of these changes in seagrass communities across the Caribbean are difficult to assess at this point because baseline information concerning the structure, processes and drivers of Caribbean seagrass beds is deficient. However, it is likely that the ecosystem services offered by the seagrass communities will be compromised by changes in their productivity and composition of the seagrass community. For example, a community shift from *T. testudinum* to faster-growing seagrass and/or algal species (observed at 7 stations, Fig. 5) will result in a change in the overall structure of the seagrass canopy, and possibly a change in associated fauna. Sediment retention is likely to be compromised as seagrass communities shift away from broad-bladed and deeply-rooted *T. testudinum*. Faster-growing seagrass species have a less developed below-ground rhizome-root system [40] which could also have negative consequences for below-ground carbon sequestration [41], or resistance of seagrass beds to hurricanes. Impacts of hurricanes are most deleterious in already disturbed beds [42], and the faster growing *S. filiforme* is much more susceptible to dislodgement than *T. testudinum* [34], [43]. The fate of the seagrass beds at Barbados-St. Lawrence lagoon (site 14) is a good example of how long-term (chronic) anthropogenic stress can act synergistically with acute extreme disturbance events [over-grazing by an exceptionally strong recruitment of sea urchins, Hurricane Ivan (2004) and Tropical Storm Emily (2005)] to cause collapse of an ecosystem. Even after 7 years, this lagoon has shown only minimal recovery, with just a few impoverished *T. testudinum* plants in areas of coral rubble and a very sparse vegetation of *Halodule wrightii* appearing in the sand areas (H. Oxenford, unpublished data).

Interpretation of the long-term shifts in the seagrass communities is not unequivocal, because responses of individual communities depend on local conditions and the state of the community when monitoring began. For example, productivity and biomass were expected to decrease with decreasing water clarity, a relationship reported for Cuba (site 4) and Costa Rica (site 20-station 49). At Belize (site 12), conflicting trends at the two stations (25 and 26) resulted from differing environmental contexts and initial states of the two seagrass meadows. At station 25, a relatively low energy site inside the lagoon, increased productivity is more likely a response to nutrient enrichment associated with the well-documented increases in turbidity [44] than to the declining light levels at this shallow (1 m) depth. Further evidence for this is that shoot density of *T. testudinum* declined by 50% from 1993 to 2012 (Fig. 5F) while the relative abundance of *S. filiforme* increased (Fig. 5B), both typical responses to declining light levels and/or nutrient enrichment. By contrast, station 26, established in 1997 in a higher-energy zone adjacent to a cut in the barrier reef-line, was scoured by Hurricane Mitch in 1998. Trends at this station largely reflect recovery of the seagrass meadow over the first 8-10 years of monitoring (Table 1).

The area of seagrass sampled by the CARICOMP protocol (0.08–0.12 m^2^ for foliar productivity and 0.04–0.09 m^2^ for biomass) is smaller than that of more recently established monitoring programs such as Seagrass Watch [45], Seagrass Monitoring in the Florida Keys National Marine Sanctuary [46], or Seagrass Net [25], that employ cover estimates of the vegetation in 5–11 quadrats (0.25–1.0 m^2^) along one to three 50m-long transects (supplemented with small samples of leaves, complete plants, sediments or seeds). Small sample size assumes a relatively homogeneous distribution of the species (or species groups). Long-living seagrasses (such as *T. testudinum*) in continuous beds, without obvious environmental gradients, may fulfil this assumption; however, a larger scale-sampling scheme (such as that of Seagrass Watch or Seagrass Net) may be necessary for less-abundant and more irregular distributed species such as the more ephemeral seagrasses (e.g *Halodule* spp. or *Halophila* spp.) or rooted macro-algae. The CARICOMP measures of foliar productivity and total plant biomass are destructive and therefore cannot be employed on a large scale (and also because sample processing is labor-intensive), but these measures are less subjective than cover estimates. In addition, they may be more sensitive to small shifts in plant abundance or biomass distribution and detect responses to environmental change sooner than monitoring programs based on less precise estimates of abundance such as vegetation cover. However, cover estimates are useful to assess loss of seagrasses over large areas under regimes of relatively severe (human-induced) stress (e.g.[25]). Thus, optimal design of a monitoring protocol depends on its goals, site- and plant characteristics and logistics. Regardless of the protocol, it is paramount for determining trends in seagrass communities that consistent observations be made over long periods (5-10 y at least). The CARICOMP program depended on voluntary participation and local resources, which resulted in large differences in sampling frequency and periods among sites. Only 17 of the 22 sites obtained sufficient data for analysis of long term trends in the seagrass communities; and even among these sites the sampling period (5–15 y, Table 1) and frequency (24–124 biomass samples, 30–202 productivity samples, Table S6) varied considerably. We may therefore, have missed possible changes at stations which were sampled infrequently or for shorter periods (5 or 6 y).

CARICOMP was a pioneering monitoring program, and this study has shown that the simple and low-cost methods used were sufficient to discern long-term trends in the seagrass communities. We suggest that changes across various parameters are consistent with deterioration of the coastal environment, thereby indicating sites that would benefit from further studies and management efforts. We recognize that drivers of change were poorly covered in this program, and suggest that future monitoring of the long-term trends should include relevant environmental measures, such as nutrient availability (C, N, P contents in plant tissues) and sediment (anoxia, organic matter) conditions, in addition to (pulse fluctuations in) water transparency, temperature and salinity. Environmental degradation often involves multiple interacting stressors, and long-term monitoring programs such as CARICOMP can only determine causal factors of change when ecological data are viewed together with supplemental data on environmental conditions.

## Supporting Information

Figure S1 **Plots of the mean monthly shoot growth rates.** Vertical bars represent 95% confidence limits. Horizontal bar above X-axis represents periods of High (black) and Low (grey) growth season. See legend Table S5 for further information. Note differences in ordinate scales for growth. +: Mean monthly SST.
(DOCX)

Figure S2 **Passage of hurricanes and storms.** Tracks of named storms or hurricanes that passed within 1 degree (60 nm or 111 km) of any CARICOMP seagrass site (indicated by the numbered circles) during the observation periods reported in Table S1. The size of the event when passing the site was not considered, thus the atmospheric and hydrological impacts at the locations may have varied from weak to severe, and other events of impact (e.g surge or excessive rains) may not be included. Servicio Académico de Monitoreo Meteorológico y Oceanográfico, Unidad Académica de Sistemas Arrecifales, Instituto de Ciencias del Mar y Limnología, Universidad Nacional Autónoma de México).
(DOCX)

Table S1 **CARICOMP seagrass monitoring sites.** General information on the sites and stations (ordered from North to South), together with sampling periods (as mm/yy) for *Thalassia testudinum* leaf productivity (Table S2) and community biomass (Table S3). Hurricanes/Storms: year of passage (′yy) and max. strength when passing the affected location (T Tropical Storm, H hurricane) in parenthesis (see Figure S2). nd not determined. British OT: British Overseas Territory.
(DOCX)

Table S2 ***Thalassia testudinum*** **leaf dynamics.** Depths (below MTL) at the stations and average values (± SE) for parameters of *T. testudinum* leaf productivity, biomass and density. N: number of samples (10×20 cm, see S1 for period). N for foliar shoot density is less at some sites because this measure was introduced later in the protocol. Secchi: mean Secchi readings from 1993-1995 (from CARICOMP, 1998).*Celestun is in the Gulf of Mexico, na: not available, nd: not determined.
(DOCX)

Table S3 **Community biomass.** Average values (± SE) of the biomass of the community by vegetation group. N: number of (core) samples (see S1 for period). Core diam: Diameter of the core samples. Total: biomass of above- and belowground live tissues. AB: above-ground biomass. Biomass of calcareous algae expressed as somatic (decalcified) weight, ∼85% of the calcified dry weight is CaCO_3_. Below-ground tissues of the algae were not considered. Biomass cores were not taken at Site 2 (Florida). “Other grass”: mostly *Syringodium filiforme* but includes *Halodule wrightii* at Station 7. * Celestun is in the Gulf of Mexico. na: not applicable.
(DOCX)

Table S4 **Intra-annual variability in** ***Thalassia testudinum*** **leaf productivity.** Results of One-sample t-test for significant differences of ΔP (deviations from general mean leaf productivity) during High- and Low-growth season at different latitudes. H0: Average ΔP = 0, α = 0.05.
(DOCX)

Table S5 **Correlations between temperature, light and** ***Thalassia testudinum*** **leaf growth.** Correlations between mean monthly SST (Sea Surface temperature, °C), H daylight (Hours of daylight) and shoot growth rates of *Thalassia testudinum* at USA-Florida Keys (Site 2, 1996–2003), Mexico-Puerto Morelos (Site 5, 1990–1991: from Van Tussenbroek BI [1995] *Thalassia testudinum* leaf dynamics in a Mexican Caribbean reef lagoon. Mar Biol 122: 33–40) and Tobago-Bon Accord Lagoon (Site 18, 1997–2007), N number of months, ns: not significant. Hours daylight were obtained from http://astro.unl.edu/classaction/animations/coordsmotion/daylighthoursexplorer.html. Mean monthly SST were from: NOAA Coral Reef Watch, Coral Bleaching Virtual Stations (http://www.osdpd.noaa.gov/ml/ocean/cb/virtual_stations.html *)-Sombrero Reef, Florida* (Site 2), Rodríguez-Martínez RE, Ruíz-Rentería F, Van Tussenbroek BI, Barba-Santos G, Escalante-Mancera E et al. [2010] State and environmental tendencies of the Puerto Morelos CARICOMP site, Mexico. Rev Biol Trop 58: 23–43 (Site 5), and R. Juman, J. Gomez [unpublished data] (Site 18).
(DOCX)

Table S6 **Regression lines of trends.** Results for the linear regressions of selected parameters *vs* year to indicate possible trends Regressions were computed for those stations and parameters when the sampling covered at least 5 y with at least six sampling events and at least 50% of the samples had values >0. *: not determined, ns: not significant, negative t indicated a negative slope. The significance level (α) is 0.05, but a Bonferroni correction is applied to this level of significance, because the parameters are derived from the same cores (Total above-ground biomass. Relative abundance of faster-growing seagrass, Relative abundance of faster growing fleshy algae, % Above-ground/total biomass for *Thalassia testudinum*) or quadrats (Productivity, Foliar shoot density of *T. testudinum*). The results of the regressions of foliar weight per shoot of *T. testudinum* is also given, to facilitate interpretations of the results, although this was not a parameter for potential degradation of the coastal environment.
(DOCX)
