# Correction: Caribbean-Wide, Long-Term Study of Seagrass Beds Reveals Local Variations, Shifts in Community Structure and Occasional Collapse

**DOI:** 10.1371/journal.pone.0098377

**Published:** 2014-05-21

**Authors:** The PLOS ONE Staff

There are errors in Table 1. The cross symbols in the “Station” column are mistakenly triplicated, and the cross symbol in the legend is displayed as question marks. The publisher apologizes for the errors. Please see the corrected Table 1 and legend here.

**Table 1:** Long-term trends at CARICOMP seagrass stations, including observations on disturbances.

Site	Station	Country/Territory (sampling period: yr-yr)	Biomass	Rel. ab. - Other seagrass	Rel. Ab. - Fleshy algae	% Above / Total Biomass	Productivity	Shoot density	Condition at beginning of monitoring	
1	1 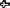	Bermuda	D	-	-	D	D	I	PRIST	The pristine seagrass beds have showed catastrophic declines since 1997, and they were extirpated in 2001, likely caused by excessive grazing by the green turtle *Chelonia mydas* ^1,2^.
	2 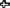	(94-02)	D	-	-	D	D	D		
2	4	USA	-	-	-	-	n	n	INT	Florida Keys suffer from eutrophication through groundwater contamination from septic tanks^3,4^; only the inshore station 5 showed indications of disturbance.
	5^*^	(96-03)	I^N^	-	-	-	I^N^	I		
3	6	Bahamas	n	n	-	n	-	-	EUTR	San Salvador had plantations, but has become a tourist destination since 1970s ^5^.
	7	(/94-06)	n	D	-	n	-	-		
4	8*	Cuba	D^T^	-	-	I^NT^	n	D^T^	PRIST	Cayo Coco was considered as pristine, but increasing sedimentation (decreased light) could have forced changes in seagrass beds^5^.
	9*	(94-02)	D^T^	-	-	I^NT^	n	D^T^		
5	10*	Mexico	n	I^NT^	I^NT^	I^NT^	I^N^	I	PRIST	Increased eutrophication through ground-water discharge likely forced changes in the seagrass beds at Puerto Morelos reef lagoon^6,7,8^. Vegetation at the coastal fringe (station 13) was buried during hurricane Wilma (2005)^7^.
	11*	(93-09)	I^N^	n	I^NT^	I^NT^	n	I		
	12*		n	I^NT^	-	I^NT^	I^N^	n		
	13* 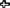		I^N^	n	n	I^NT^	n	n		
7	15	Cayman I.	-	-	-	-	I^N^	D^T^	INT	The high number of visitors to the Grand Cayman compromise carrying capacity and cause environmental degradation, but not in protected CARICOMP areas^9^.
		(97-03)								
8	17*	Jamaica	I^N^	-	-	I^NT^	I^N^	-	DIST	Discovery Bay has been affected by terrestrial runoff from agricultural developments and possibly by proliferation of urban developments without a central sewage system^9^.
		(93-99)								
10	21*	Puerto Rico	I^N^	-	I^NT^	I^NT^	n	n	INT	Residential and tourism developments at and near La Parguera have increased terrigenous sediment load, and raw- and secondary sewage effluents from a treatment plant^9^ possibly changed the seagrass community at station 21.
	22	(94-06)	n	-	n	I^NT^	n	n		
12	25*	Belize	n	I^NT^	n	I^NT^	I^N^	D^T^	PRIST	Both sites have been subjected to loss of water clarity due to increased input of sediments and nutrients from coastal development and agriculture. *T. testudinum* shoot density has declined at Site 25(Twin Cays) possibly due to increased sedimentation^10^. Station 26 (Carrie Bow Cay) was scoured by Hurricane Mitch (1998) but recovering^11^.
	26	(93-12)(97-12)	n	n	n	I^NT^	n	I		
13	29	Isla	n	n	-	n	n	n	PRIST	Isla Providencia (Columbia) was for a long time sparsely inhabited; recently tourism has increased, but the changes in seagrass beds were not consistent with environmental degradation. Hurricane Beta passed close by, but the seagrasses received no impact^12.^
	30	Providencia	n	D	-	n	n	n		
	31	(00-07)	n	n	-	n	D^T^	n		
14	33* 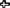	Barbados	n	I^NT^	-	I^NT^	n	D^T^	DIST	St. Lawrence has been affected by anthropogenic activities since 1880s from sugar cane cultivation, residential developments and eutrophication. Combined effects of increased sedimentation due to frequent flushing of a new sewage pipe system, excessive sea urchin grazing and storms caused collapse of the seagrass beds^5, 13^.
	34* 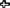	(93-01)	n	I^NT^	-	I^NT^	n	-		
15	37	San Andres	n	n	-	n	-	I	DIST	San Andres Island (Colombia) is the most densely populated oceanic island in the Caribbean. Past population increase with poorly planned development resulted in degradation of coastal ecosystems^5^ The seagrass community did not show changes.
	38	(99-07)	n	n	-	n	-	n		
17	41	Colombia	n	-	-	n	n	n	PRIST	Seagrass beds, mangroves and coral reefs at Chengue Bay are healthy and stable^14^. Abundant *Halimeda opuntia* before 1996 disappeared without obvious cause^5^.
	42	(94-05)	n	-	-	n	n	n		
18	43	Tobago	n	-	-	n	n	n	DIST	Bon Accord Lagoon has received impacts from tourism and sewage for ∼ 50y^9^. But apart from a decrease in productivity at station 45, the seagrass conditions were stable.
	44	(92-07)	n	-	-	n	D^T^	n		
20	47*	Venezuela	I^N^	-	-	I^NT^	I^N^	I	DIST	Morrocoy Park has been subjected to increasing land-based constructions, mangrove demolition and sewage effluents since 1970s^9^. Heavy rainfall in 1999 caused loss of seagrass leaves, but recovery followed within months^5^.
	48*	(93-06)	I^N^	-	-	I^NT^	I^N^	I		
21	49*	Costa Rica	D^T^	-	-	n	D^T^	D^T^	PRIST	The Limón earthquake in 1991 affected the seagrass beds at Cahuita which fully recovered after 1y^5^. Seagrass beds at Station 49 may have deteriorated due to increased turbidity by sewage load, sediments and fertilizers (citrus and banana farming)^15, 16^.
	50	(99-05)	-	-	-	-	D^T^	n		
22	51	Panama	I^N^	-	-	-	n	n	DIST^16^	The narrow *Thalassia testudinum* bed fringing a mangrove swamp showed increased biomass over the years, which may be attributed to natural causes^17^.
	52	(99-06)	I^N^	-	-	-	n	I		

Biomass: total above-ground biomass of the community; Rel abund: relative abundance (biomass) of faster growing seagrass and algal species; Other seagrass: seagrass species other than *Thalassia testudinum* (mostly *Syringodium filiforme*); % Above / Total Biomass: percentage of above-ground of total biomass of *T. testudinum* (*S. filiforme* for site 14, because *T. testudinum* was absent in later years at station 33); Productivity: productivity of leaves of *T. testudinum*. 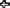 Collapse of seagrass bed, * seagrass beds showed changes that potentially indicate with human-induced environmental deterioration. Trends: **I** increase, **D** decrease, n without change, - not determined, **^N^** expected change due to increasing nutrient load, **^T^** expected change due to increasing turbidity, **^NT^** expected change due to either increasing turbidity or nutrient load, changes without symbol were not consistent with expectations of water quality deterioration (See text for further explanation). Conditions at the beginning of monitoring: PRIST (relatively) pristine (undisturbed by humans); INT Moderate disturbance; DIST Disturbed (eutrophication, terrestrial runoff, or overfishing, from [26], [29], [47]). See Table S6 for information on regression lines. Source: 1. [48], 2. [49], 3. [50], 4. [51], 5. [29], 6. [52], 7. [33], 8. [53], 9. [38], 10. [44], 11. Pers. Obs. K. Koltes, 12.Pers.Obs. H.A Oxenford, 13. [54], 14. [55], 15. [56], 16. [57], 17. [58].
