# Supplementary material for: Caribbean-Wide, Long-Term Study of Seagrass Beds Reveals Local Variations, Shifts in Community Structure and Occasional Collapse
Source: PLoS One. 2014 Mar 3;9(3):e90600. doi: 10.1371/journal.pone.0090600 (PMC4036797; doi:10.1371/journal.pone.0090600)
Supplement: Figure S1 — Plots of the mean monthly shoot growth rates. Vertical bars represent 95% confidence limits. Horizontal bar above X-axis represents periods of High (black) and Low (grey) growth season. See legend Table S5 for further information. Note differences in ordinate scales for growth. +: Mean monthly SST. (DOCX) [file pone.0090600.s001.docx]

**Figure S1.**

**Plots of the mean monthly shoot growth rates.**

Vertical bars represent 95% confidence limits. Horizontal bar above X-axis represents periods of High (black) and Low (grey) growth season. See legend Table S5 for further information. Note differences in ordinate scales for growth. +: Mean monthly SST.

**
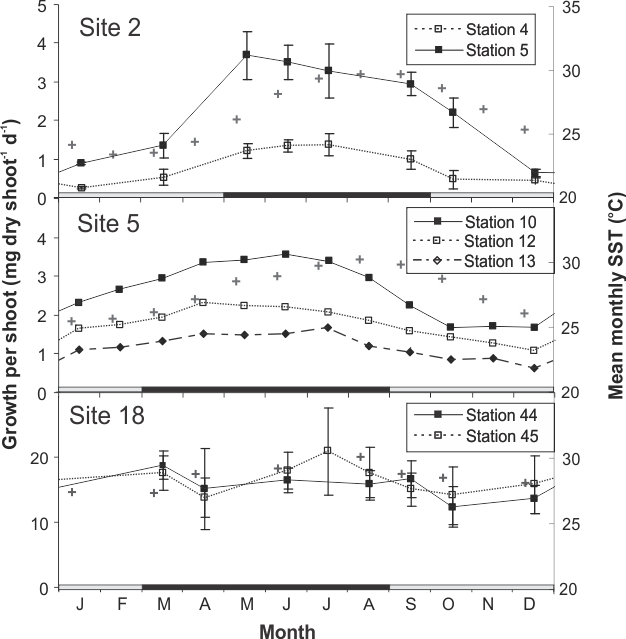
**
