# Supplementary material for: Caribbean-Wide, Long-Term Study of Seagrass Beds Reveals Local Variations, Shifts in Community Structure and Occasional Collapse
Source: PLoS One. 2014 Mar 3;9(3):e90600. doi: 10.1371/journal.pone.0090600 (PMC4036797; doi:10.1371/journal.pone.0090600)

**Figure S2.**

**Passage of hurricanes and storms.**

Tracks of named storms or hurricanes that passed within 1 degree (60 nm or 111 km) of any CARICOMP seagrass site (indicated by the numbered circles) during the observation periods reported in Table S1. The size of the event when passing the site was not considered, thus the atmospheric and hydrological impacts at the locations may have varied from weak to severe, and other events of impact (e.g surge or excessive rains) may not be included. Servicio Académico de Monitoreo Meteorológico y Oceanográfico, Unidad Académica de Sistemas Arrecifales, Instituto de Ciencias del Mar y Limnología, Universidad Nacional Autónoma de México).


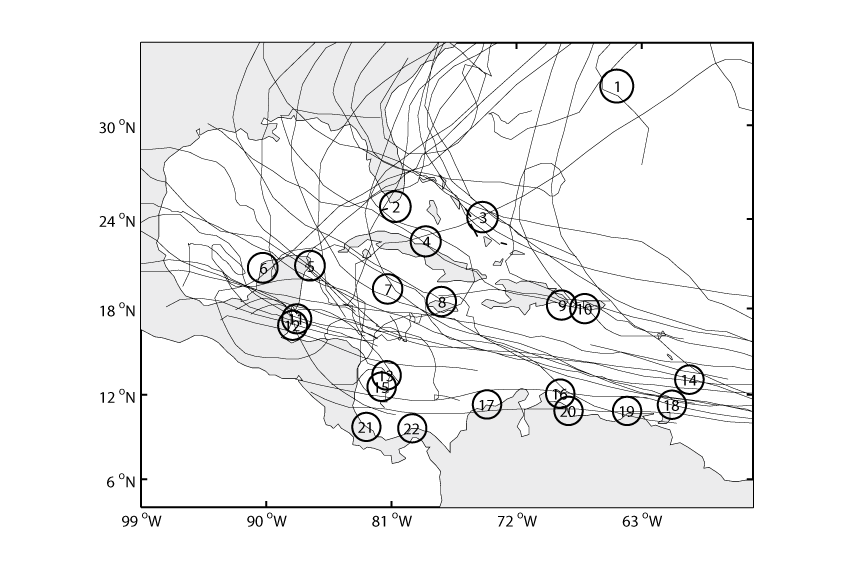

Supplement: Figure S2 — Passage of hurricanes and storms. Tracks of named storms or hurricanes that passed within 1 degree (60 nm or 111 km) of any CARICOMP seagrass site (indicated by the numbered circles) during the observation periods reported in Table S1. The size of the event when passing the site was not considered, thus the atmospheric and hydrological impacts at the locations may have varied from weak to severe, and other events of impact (e.g surge or excessive rains) may not be included. Servicio Académico de Monitoreo Meteorológico y Oceanográfico, Unidad Académica de Sistemas Arrecifales, Instituto de Ciencias del Mar y Limnología, Universidad Nacional Autónoma de México). (DOCX) [file pone.0090600.s002.docx]
