# Supplementary material for: Caribbean-Wide, Long-Term Study of Seagrass Beds Reveals Local Variations, Shifts in Community Structure and Occasional Collapse
Source: PLoS One. 2014 Mar 3;9(3):e90600. doi: 10.1371/journal.pone.0090600 (PMC4036797; doi:10.1371/journal.pone.0090600)
Supplement: Table S1 — CARICOMP seagrass monitoring sites. General information on the sites and stations (ordered from North to South), together with sampling periods (as mm/yy) for Thalassia testudinum leaf productivity (Table S2) and community biomass (Table S3). Hurricanes/Storms: year of passage (′yy) and max. strength when passing the affected location (T Tropical Storm, H hurricane) in parenthesis (see Figure S2). nd not determined. British OT: British Overseas Territory. (DOCX) [file pone.0090600.s003.docx]

**Table S1.** CARICOMP seagrass monitoring sites.

General information on the sites and stations (ordered from North to South), together with sampling periods (as mm/yy) for *Thalassia testudinum* leaf productivity (Table S2) and community biomass (Table S3). Hurricanes/Storms: year of passage (´yy) and max. strength when passing the affected location (T Tropical Storm, H hurricane) in parenthesis (see Figure S2). nd not determined. British OT: British Overseas Territory.

| **Site** | **Sta-** | **Country/** | **Site** | **Local Name** | **Lat.** | **Long.** | **Productivity** | **Biomass** | | **Hurricanes/Storms** | |  |
| --- | --- | --- | --- | --- | --- | --- | --- | --- | --- | --- | --- | --- |
|  | **tion** | **Territory** |  |  | **(°N)** | **(°W)** | **(Period)** | **(Period)** | |  | |  |
| **1** | **1** | British OT | Bermuda | N Seagrass | 32°24´ | 64°48´ | 04/94-06/02 | | 04/93-06/99 | | Felix (´95^1.^), Gert (’99^1.^), | |
|  | **2** |  |  | S Seagrass |  |  | 05/93-02/99 | | 05/93-06/99 | | Florence (´00 H1), Karen (´01 | |
|  | **3** |  |  | W Seagrass | 32°24´ | 64°48´ | 02/00-06/00 | | 07/00 | | T) | |
| **2** | **4** | USA | Florida Keys | Long Key I | 24°48´ | 80°43´ | 01/96-03/03 | | nd | | George (´98 H2), Irene (´99 H1) | |
|  | **5** |  |  | Long Key II | 24°48´ | 80°49´ | 01/96-03/03 | | nd | |  | |
| **3** | **6** | Bahamas | San Salvador | Grahams Harbour | 24°07´ | 74°27´ | 07/94-05/06 | | 07/94-05/06 | | Erin(´95 H1), Bertha (´96 H1), Lili (´96 H3), Denis (´99 H1), | |
|  | **7** |  |  | French Bay | 23°57´ | 74°31´ | 07/94-05/06 | | 07/94-05/06 | | Floyd (´99 H4), Frances (´04 H4) | |
| **4** | **8** | Cuba | Cayo Coco | Seagrass A | 22°31´ | 78°32´ | 03/94-03/02 | | 03/94-03/02 | | Gordon (´94 T), Lili (´96 H2), | |
|  | **9** |  |  | Seagrass B | 22°31´ | 78°32´ | 03/94-03/02 | | 03/94-03/02 | | George (’98 H1) | |
| **5** | **10** | Mexico | Pto. Morelos | High Prod | 20°54´ | 86°51´ | 02/93-06/09 | | 02/93-06/09 | | Alison (´95 T), Roxanne (´95 H3), Isidore (`02-H3), Claudette (´03 T), Emily (´05 H4), Ivan (´04^2.^), Wilma (´05 H4), Dolly (´08 T) | |
|  | **11** |  |  | Typical | 20°52´ | 86°52´ | 02/93-06/09 | | 02/93-06/09 | |  |  |
|  | **12** |  |  | Reef | 20°52´ | 86°51´ | 02/93-06/09 | | 02/93-06/09 | |  |  |
|  | **13** |  |  | Coast | 20°52´ | 86°52´ | 02/93-07/05 | | 02/93-07/05 | |  |  |
| **6** | **14** | Mexico | Celestun^1.^ | Celestun | 20°45´ | 90°15´ | 06/95 | | 09/94-07/95 | | Opal (´95 T) | |
| **7** | **15** | Cayman | Grand Cayman | North Sound 1 | 19°19´ | 81°16´ | 03/97-05/03 | | 03/97-05/03 | | Isidore (´02 T), Lili (’02 H1) | |
|  | **16** | Islands | Grand Cayman | North Sound 2 | 19°19´ | 81°16´ | 09/99-05/03 | | 09/99-05/03 | |  | |
| **8** | **17** | Jamaica | Discovery Bay | Discovery Bay A | 18°28´ | 77°24´ | 02/93-01/99 | | 02/93-01/99 | | Gordon (´94 T) | |
|  | **18** |  |  | Discovery Bay B | 18°28´ | 77°24´ | 02/93-01/99 | | 02/93-01/99 | |  | |
| **9** | **19** | Dominican | P. Nac. Este | H. los Cocos 1 | 18°14´ | 68°46´ | 03/96-09/01 | | 03/96-09/01 | | Hortense (´96 H1), | |
|  | **20** | Republic |  | H. los Cocos 2 | 18°14´ | 68°46´ | 03/96-10/96 | | 03/96-02/00 | | George´s (´98 H3) | |
| **10** | **21** | Puerto Rico | La Parguera | Caballo Blanco | 17°59´ | 67°03´ | 10/98-09/07 | | 09/94-09/07 | | Hortense (´96 H1), | |
|  | **22** |  |  | Enrique | 17°56´ | 67°03´ | 10/98-09/07 | | 09/94-09/07 | | George´s (´98 H2), Jeanne (’04 T) | |
| **11** | **23** | Belize | Turneffe Isl. | Calabash Cay 1 | 17°17´ | 87°49´ | 01/98 | | 10/96 | |  | |
|  | **24** |  |  | Calabash Cay 2 | 17°17´ | 87°49´ | 01/98 | | 10/96 | |  | |
| **12** | **25** | Belize | Twin Cays |  | 16°50´ | 88°06´ | 08/93-12/12 | | 08/93-12/12 | | Gert (´93 T), Mitch (´98^3.^), Keith | |
|  | **26** |  | Carrie Bow  Cay |  | 16°48´ | 88°05´ | 01/97-12/12 | | 01/97-12/12 | | (´00 H3), Iris (´01 H4), Arthur (´08 T), Richard (’10 H2),Harvey (´11 T) | |
| **13** | **27** | Colombia | I. Providencia | Camp N | 13°19´ | 81°06´ | 06/00-03/07 | | 06/00-03/07 | | Beta (´05 H1) | |
|  | **28** |  |  | Camp S | 13°19´ | 81°06´ | 06/00-03/07 | | 06/00-03/07 | |  | |
|  | **29** | Colombia | I. Providencia | Fort N | 13°19´ | 81°06´ | 03/00-03/07 | | 03/00-03/07 | |  | |
|  | **30** |  |  | Fort S | 13°19´ | 81°06´ | 03/00-03/07 | | 03/00-03/07 | |  | |
|  | **31** | Colombia | I. Providencia | McBean N | 13°19´ | 81°06´ | 06/00-03/07 | | 06/00-03/07 | |  | |
|  | **32** |  |  | McBean S | 13°19´ | 81°06´ | 06/00-03/07 | | 06/00-03/07 | |  | |
| **14** | **33** | Barbados | St. Lawrence | St. Lawrence L. E | 13°04´ | 59°35´ | 04/93-02/01 | | 04/93-09/01 | | Debby (´94 T), Marilyn (´95 H1), | |
|  | **34** |  | Lagoon | St. Lawrence L. W | 13°04´ | 59°35´ | 04/93-02/01 | | 04/93-09/01 | | Chantal (’01 T), Lili (’02 T) | |
| **15** | **35** | Colombia | I. San Andrés | Sprat Bight E | 12°35´ | 81°42´ | 05/02-12/02 | | nd | | Beta (´05 T) | |
|  | **36** |  |  | Sprat Bight W | 12°35´ | 81°42´ | 05/02-12/02 | | nd | |  | |
|  | **37** | Colombia | I. San Andrés | Cotton Cay N | 13°19´ | 81°21´ | 04/99-03/07 | | 04/99-03/07 | |  | |
|  | **38** |  |  | Cotton Cay S | 12°28´ | 81°40´ | 04/99-03/07 | | 04/99-03/07 | |  | |
| **16** | **39** | Curaçao | Spaanse Water | Spaanse Water 1 | 12°05´ | 68°52´ | 03/94-07/98 | | 03/94-09/95 | | Cesar (´96 T) | |
|  | **40** |  |  | Spaanse Water 2 | 12°05´ | 68°52´ | 01/98-07/98 | | nd | |  | |
| **17** | **41** | Colombia | Chengue Bay | Chengue Bay 1 | 11°19´ | 74°08´ | 03/94-08/05 | | 03/94-08/05 | | Cesar (´96 T), Lenny (´99^4.^) | |
|  | **42** |  |  | Chengue Bay 2 | 11°19´ | 74°08´ | 03/94-08/05 | | 03/94-08/05 | |  | |
| **18** | **43** | Tobago | Bon Accord L. | Bon Accord L. I | 11°18´ | 60°50´ | 03/93-06/07 | | 10/92-03/07 | | Bret (´93 T), Joyce (’00 T), | |
|  | **44** |  |  | Bon Accord L. II | 11°18´ | 60°50´ | 10/96-06/07 | | 04/97-03/07 | | Earl (´04 T), Ivan (´04 H3), | |
|  | **45** |  | Pigeon Point | Pigeon Point | 11°18´ | 60°83´ | 03/00-06/00 | | 03/00 | | Emily (´05 H1) | |
| **19** | **46** | Venezuela | I. de Margarita | Punta Mangle | 10°52´ | 64°03´ | 11/95-05/97 | | nd | | Cesar (´96 T) | |
| **20** | **47** | Venezuela | Morrocoy | Las Luisas | 10°52´ | 68°16´ | 06/93-09/06 | | 03/93-10/06 | | Bret (’93 T) | |
|  | **48** |  |  | Tumba Cuatro | 10°52´ | 68°16´ | 03/94-09/06 | | 03/94-10/06 | |  | |
| **21** | **49** | Costa Rica | Cahuita | Rio Perezozo 1 | 9°44´ | 82°48´ | 03/99-01/05 | | 04/99-03/04 | |  | |
|  | **50** |  |  | Rio Perezozo 2 | 9°44´ | 82°48´ | 03/99-03/03 | | 04/99-03/03 | |  | |
| **22** | **51** | Panama | I. de Colon | STRI_Colo 1 | 9°21´ | 82°15´ | 03/99-07/12 | | 03/99-07/12 | |  | |
|  | **52** |  |  | STRI_Colo 2 | 9°21´ | 82°15´ | 03/99-07/12 | | 03/99-07/12 | |  | |

1-4. Hurricanes that did not pass the site closely, but caused an impact on its coastal areas. 1. Pers. Obs. R. Smith, 2. Pers. Obs. B.I. van Tussenbroek, 3. Koltes KH, Opishinski T (2009) Patterns of water quality and movement in the vicinity of Carrie Bow Cay, Belize. Smith Contr Mar Sc 38: 379–390., 4. Rodríguez-Martínez RE, Ruíz-Rentería F, Van Tussenbroek BI, Barba-Santos G, Escalante-Mancera E et al. (2010) State and environmental tendencies of the Puerto Morelos CARICOMP site, Mexico. Rev Biol Trop 58 (Suppl. 3): 23-43.
