# Supplementary material for: Caribbean-Wide, Long-Term Study of Seagrass Beds Reveals Local Variations, Shifts in Community Structure and Occasional Collapse
Source: PLoS One. 2014 Mar 3;9(3):e90600. doi: 10.1371/journal.pone.0090600 (PMC4036797; doi:10.1371/journal.pone.0090600)
Supplement: Table S2 — Thalassia testudinum leaf dynamics. Depths (below MTL) at the stations and average values (± SE) for parameters of T. testudinum leaf productivity, biomass and density. N: number of samples (10×20 cm, see S1 for period). N for foliar shoot density is less at some sites because this measure was introduced later in the protocol. Secchi: mean Secchi readings from 1993-1995 (from CARICOMP, 1998).*Celestun is in the Gulf of Mexico, na: not available, nd: not determined. (DOCX) [file pone.0090600.s004.docx]

**Table S2**

***Thalassia testudinum* dynamics.**

Depths (below MTL) at the stations and average values (± SE) for parameters of *T. testudinum* leaf productivity, biomass and density. N: number of samples (10 x 20cm, see S1 for period). N for foliar shoot density is less at some sites because this measure was introduced later in the protocol. Secchi: mean Secchi readings from 1993-1995 (from CARICOMP, 1998).*Celestun is in the Gulf of Mexico, na: not available, nd: not determined.

| **Site** | **Sta-tion** | **Country/**  **Territory** | **Depth (m)** | **Secchi (m)** | **N** | **P - Leaf Productivity** | **B - Leaf**  **Biomass** | **P/B ratio**  **( % d^-1^)** | **Foliar shoot density** | **Foliar shoot weight** | **N**  **Foliar shoot** |
| --- | --- | --- | --- | --- | --- | --- | --- | --- | --- | --- | --- |
|  |  |  |  |  |  | **(g dry m^-2^ d^-1^)** | **(g dry m^-2^)** |  | **(no m^-2^)** | **(mg)** | **density** |
| **1** | **1** | Bermuda | 5.0 | 14.4 | 88 | 0.92 ± 0.06 | 26.70 ± 1.71 | 3.77 ± 0.12 | 702.6 ± 38.9 | 36.7 ± 3.2 | 76 |
|  | **2** |  | 5.0 |  | 75 | 0.82 ± 0.06 | 22.17 ± 1.92 | 4.07 ± 0.13 | 602.3 ± 25.7 | 30.3 ± 2.7 | 64 |
|  | **3** |  | 5.0 |  | 8 | 0.08 ± 0.01 | 1.52 ± 0.17 | 5.04 ± 0.33 | 368.8 ± 49.0 | 4.9 ± 0.9 | 8 |
| **2** | **4** | USA | 3.1 | na | 168 | 0.52 ± 0.04 | 25.95 ± 0.91 | 1.88 ± 0.09 | 568.5 ± 15.2 | 46.7 ± 1.3 | 168 |
|  | **5** |  | 7.1 |  | 165 | 0.84 ± 0.05 | 50.84 ± 2.57 | 1.65 ± 0.05 | 387.0 ± 11.9 | 130.3 ± 5.0 | 165 |
| **3** | **6** | Bahamas | 3.0 | 12.2 | 31 | 1.01 ± 0.12 | 28.19 ± 2.48 | 3.46 ± 0.25 | nd | nd | nd |
|  | **7** |  | 2.0 |  | 33 | 1.43 ± 0.27 | 38.84 ± 6.43 | 3.65 ± 0.24 | nd | nd | nd |
| **4** | **8** | Cuba | 1.4 |  | 96 | 2.03 ± 0.09 | 80.74 ± 3.51 | 2.73 ± 0.11 | 663.3 ± 27.3 | 133.5 ± 6.8 | 83 |
|  | **9** |  | 1.8 |  | 102 | 1.84 ± 0.06 | 78.32 ± 2.80 | 2.51 ± 0.08 | 656.7 ± 22.4 | 130.9 ± 5.8 | 90 |
| **5** | **10** | Mexico | 3.5 | 15.0 | 178 | 1.69 ± 0.05 | 73.88 ± 2.42 | 2.34 ± 0.04 | 542.5 ± 12.5 | 138.5 ± 3.7 | 178 |
|  | **11** |  | 2.8 |  | 174 | 0.95 ± 0.03 | 35.71 ± 1.17 | 2.70 ± 0.04 | 516.7 ± 14.1 | 72.5 ± 2.3 | 174 |
|  | **12** |  | 3.0 |  | 164 | 0.97 ± 0.03 | 31.94 ± 0.95 | 3.08 ± 0.05 | 732.1 ± 15.8 | 44.7 ± 1.3 | 164 |
|  | **13** |  | 2.4 |  | 140 | 1.51 ± 0.05 | 59.17 ± 2.13 | 2.60 ± 0.04 | 541.1 ± 14.5 | 111.4 ± 3.3 | 140 |
| **6*** | **14** | Mexico | 2.0 | na | 4 | 7.56 ± 2.81 | 165.00 ± 35.00 | 4.30 ± 0.70 | nd | nd | nd |
| **7** | **15** | Cayman Isl. | 1.5-2.5 | na | 36 | 2.97 ± 0.24 | 85.10 ± 4.43 | 3.53 ± 0.26 | 850.0 ± 42.6 | 104.8 ± 5.6 | 36 |
|  | **16** |  | 1.5-2.5 |  | 24 | 2.67 ± 0.32 | 55.06 ± 4.24 | 4.77 ± 0.27 | 1170 ± 80.8 | 47.7 ± 2.0 | 24 |
| **8** | **17** | Jamaica | 1.0 | 11.5 | 35 | 3.24 ± 0.28 | 112.50 ± 12.34 | 3.42 ± 0.32 | 523.7 ± 57.0 | 203.7 ± 21.1 | 18 |
|  | **18** |  | 1.0 |  | 29 | 4.25 ± 0.48 | 146.79 ± 17.56 | 3.15 ± 0.17 | 729.4 ± 70.7 | 311.7 ± 40.5 | 12 |
| **9** | **19** | Dominican R. | 2.0-4.0 | na | 18 | 3.15 ± 0.52 | 62.97 ± 5.92 | 5.12 ± 0.64 | 408.5 ± 24.5 | 413.2 ± 70.0 | 12 |
|  | **20** |  | 2.0-4.0 |  | 11 | 4.30 ± 1.14 | 77.27 ± 9.14 | 5.06 ± 1.05 | 375.0 ± 60.2 | 189.0 ± 24.0 | 6 |
| **10** | **21** | Puerto Rico | 1.0 | 8.3 | 48 | 5.71 ± 0.35 | 102.44 ± 6.58 | 5.76 ± 0.17 | 927.1 ± 50.7 | 122.8 ± 8.7 | 48 |
|  | **22** |  | 1.5 |  | 48 | 5.99 ± 0.28 | 105.26 ± 5.22 | 5.86 ± 0.22 | 1052.1 ± 40.6 | 105.3 ± 6.4 | 48 |
| **11** | **23** | Belize | 0.5-1.0 | na | 6 | 5.10 ± 0.45 | 146.75 ± 13.70 | 3.49 ± 0.12 | nd | 120.4 ± 13.9 | nd |
|  | **24** |  | 2.0-3.0 |  | 6 | 3.03 ± 0.27 | 79.25 ± 7.67 | 3.87 ± 0.18 | nd | 90.6 ± 15.7 | Nd |
| **12** | **25** | Belize | 1.2-1.5 | 10.8 | 168 | 3.02 ± 0.09 | 117.70 ± 3.01 | 2.58 ± 0.05 | 772.5 ± 17.0 | 162.5 ± 4.8 | 156 |
|  | **26** |  | 1.5 |  | 116 | 2.35 ± 0.06 | 82.08 ± 2.25 | 2.92 ± 0.04 | 853.0 ± 17.5 | 98.3 ± 2.6 | 116 |
| **13** | **27** | Colombia | na | na | 30 | 1.91 ± 0.18 | 66.33 ± 4.30 | 2.90 ± 0.20 | 568.3 ± 46.0 | 141.0 ± 14.0 | 30 |
|  | **28** |  | na |  | 30 | 2.27 ± 0.20 | 75.15 ± 5.10 | 2.94 ± 0.82 | 703.3 ± 52.0 | 120.9 ± 10.7 | 30 |
|  | **29** | Colombia | na |  | 35 | 1.56 ± 0.16 | 49.58 ± 4.77 | 3.13 ± 1.21 | 342.9 ± 25.7 | 145.4 ± 10.0 | 35 |
|  | **30** |  | na |  | 35 | 1.21 ± 0.11 | 43.90 ± 3.48 | 2.72 ± 0.87 | 362.9 ± 21.3 | 124.7 ± 8.5 | 35 |
|  | **31** | Colombia | na |  | 30 | 1.77 ± 0.27 | 55.85 ± 5.16 | 3.01 ± 0.27 | 360.0 ± 31.11 | 195.8 ± 25.3 | 30 |
|  | **32** |  | na |  | 30 | 2.22 ± 0.30 | 71.18 ± 7.29 | 2.96 ± 0.23 | 395.0 ± 36.6 | 221.7 ± 29.5 | 30 |
| **14** | **33** | Barbados | 1.0-3.0^1.^ | 4.5 | 64 | 2.85 ± 0.12 | 78.42 ± 3.23 | 3.75 ± 0.14 | 537.5 ± 43.2 | 149.9 ± 15.2 | 28 |
|  | **34** |  | 1.0-3.0^1.^ |  | 56 | 3.62 ± 0.23 | 97.67 ± 6.23 | 3.82 ± 0.14 | 497.9 ± 46.7 | 297.1 ± 46.9 | 24 |
| **15** | **35** | Colombia | na | 5.2 | 12 | 1.86 ± 0.39 | 77.40 ± 15.81 | 2.45 ± 0.14 | 837.5 ± 76.2 | 108.4 ± 24.2 | 12 |
|  | **36** |  | na |  | 12 | 3.96 ± 0.49 | 99.31 ± 10.61 | 3.96 ± 0.22 | 881.3 ± 46.2 | 107.0 ± 20.7 | 8 |
|  | **37** | Colombia | na |  | 40 | 2.84 ± 0.32 | 85.51 ± 12.52 | 3.81 ± 0.31 | 697.6 ± 51.6 | 155.9 ± 22.1 | 40 |
|  | **38** |  | na |  | 38 | 2.16 ± 0.23 | 71.75 ± 6.30 | 3.31 ± 0.25 | 618.4 ± 31.6 | 123.7 ± 11.4 | 38 |
| **16** | **39** | Curaçao | 1.0-2.0 | na | 30 | 1.14 ± 0.17 | 46.70 ± 6.72 | 3.21 ± 0.26 | 250.0 ± 12.8 | 331.1 ± 68.4 | 24 |
|  | **40** |  | 1.0-2.0 |  | 12 | 1.67 ± 0.37 | 41.17 ± 8.64 | 3.96 ± 0.25 | 258.3 ± 20.3 | 276.1 ± 59.8 | 12 |
| **17** | **41** | Colombia | 1.5 | 6.0^2.^ | 72 | 2.77 ± 0.11 | 80.40 ± 3.31 | 3.52 ± 0.09 | 630.6 ± 16.33 | 129.6 ± 4.4 | 72 |
|  | **42** |  | 1.5 |  | 72 | 2.64 ± 0.10 | 79.82 ± 6.43 | 3.47 ± 0.11 | 740.3 ± 23.19 | 109.4 ± 4.2 | 72 |
| **18** | **43** | Tobago | 0.5-1.2 | 6.6 | 202 | 3.18 ± 0.11 | 84.54 ± 3.01 | 4.00 ± 0.09 | 215.4 ± 6.8 | 433.6 ± 15.7 | 174 |
|  | **44** |  | 0.5-1.2 |  | 182 | 3.09 ± 0.13 | 79.21 ± 3.54 | 4.19 ± 0.11 | 196.1 ± 6.9 | 433.3 ± 16.4 | 169 |
|  | **45** |  | 0.5-1.2 |  | 12 | 6.07 ± 0.91 | 109.87 ± 14.15 | 5.49 ± 0.40 | 212.5 ± 24.7 | 521.5 ± 52.4 | 12 |
| **19** | **46** | Venezuela | 0.4-1.0 | 3.4 | 12 | 2.82 ± 0.43 | 64.29 ± 11.18 | 4.62 ± 0.23 | 445.8 ± 31.7 | 146.4 ± 28.5 | 12 |
| **20** | **47** | Venezuela | 1.2 | 7.4 | 60 | 4.15 ± 0.20 | 119.50 ± 5.52 | 3.11 ± 0.13 | 495.4 ± 87.1 | 258.2 ± 28.5 | 25 |
|  | **48** |  | 1.2 |  | 42 | 4.39 ± 0.26 | 114.61 ± 7.61 | 3.47 ± 0.13 | 518.2 ± 65.2 | 262.0 ± 31.8 | 18 |
| **21** | **49** | Costa Rica | 1.0 | 2.7^3.^ | 107 | 2.08 ± 0.10 | 50.31 ± 2.94 | 4.47 ± 0.12 | 934.5 ± 30.6 | 41.6 ± 2.3 | 107 |
|  | **50** |  | 1.0 |  | 74 | 2.94 ± 0.16 | 54.08 ± 4.36 | 5.48 ± 0.30 | 1078.2 ± 24.5 | 47.0 ± 5.6 | 74 |
| **22** | **51** | Panama | 2.0 | 7.9^4.^ | 162 | 1.43 ± 0.04 | 61.29 ± 1.45 | 2.37 ± 0.05 | 997.8 ± 20.4 | 63.9 ± 1.6 | 162 |
|  | **52** |  | 2.0 |  | 165 | 1.51 ± 0.04 | 76.31 ± 1.78 | 1. ± 0.03 | 867.2 ± 22.5 | 94.1 ± 2.3 | 165 |

1. Depths have varied significantly over the years as sedimentation regimes were changing (pers. Com. Hazel Oxenford), in addition tidal differences are larger in Barbados than most other areas in the Caribbean (difference between MLLW and MHHW ~0.9m), 2. Average 1996-2008: from Rodriguez-Ramírez A, Garzón-Ferreira J, Batista-Morales A, Gil DL, Gómez-López DI et al. (2010) Temporal patterns in coral reef, seagrass and mangrove communities from Chengue bay CARICOMP site (Colombia): 1993-2008. Rev Biol Trop 58 (Suppl. 3): 45-62, 3. CARICOMP (2004) Caribbean Coastal Marine Productivity Program: 1993-2003*,* Eds. Linton D, Fisher T, CARICOMP Program, 88 p., 4. Average 1999-2000 from R. Collin (unpublished data).
