# Supplementary material for: Caribbean-Wide, Long-Term Study of Seagrass Beds Reveals Local Variations, Shifts in Community Structure and Occasional Collapse
Source: PLoS One. 2014 Mar 3;9(3):e90600. doi: 10.1371/journal.pone.0090600 (PMC4036797; doi:10.1371/journal.pone.0090600)
Supplement: Table S3 — Community biomass. Average values (± SE) of the biomass of the community by vegetation group. N: number of (core) samples (see S1 for period). Core diam: Diameter of the core samples. Total: biomass of above- and belowground live tissues. AB: above-ground biomass. Biomass of calcareous algae expressed as somatic (decalcified) weight, ∼85% of the calcified dry weight is CaCO3. Below-ground tissues of the algae were not considered. Biomass cores were not taken at Site 2 (Florida). “Other grass”: mostly Syringodium filiforme but includes Halodule wrightii at Station 7. * Celestun is in the Gulf of Mexico. na: not applicable. (DOCX) [file pone.0090600.s005.docx]

**Table S3.**

**Community biomass.**

Average values (± SE) of the biomass of the community by vegetation group. N: number of (core) samples (see S1 for period). Core diam: Diameter of the core samples. Total: biomass of above- and belowground live tissues. AB: above-ground biomass. Biomass of calcareous algae expressed as somatic (decalcified) weight, ~85% of the calcified dry weight is CaCO_3_. Below-ground tissues of the algae were not considered. Biomass cores were not taken at Site 2 (Florida). “Other grass”: mostly *Syringodium filiforme* but includes *Halodule wrightii* at Station 7. * Celestun is in the Gulf of Mexico. na: not applicable.

|  |  |  |  | |  | | ***Thalassia testudinum*** | | **Other grass** | | **Algae (Above-ground)** | | **Community** | |
| --- | --- | --- | --- | --- | --- | --- | --- | --- | --- | --- | --- | --- | --- | --- |
| **Site** | **Sta-tion** | **Country/**  **Territory** | **N** | **Core diam** | | **Total** | | **AB/**  **Total** | **Total** | **AB/**  **Total** | **Calcareous** | **Fleshy** | **AB** | **Total** |
|  |  |  |  | **(cm)** | | **(g dry m^-2^)** | | **(%)** | **(g dry m^-2^)** | **(%)** | **(g dry m^-2^)** | **(g dry m^-2^)** | **(g dry m^-2^)** | **(g dry m^-2^)** |
| **1** | **1** | Bermuda | 37 | 15.4 | | 506.1 ± 44.9 | | 3.05 ± 0.35 | 0.0 | na | 0.00 | 0.0 | 15.4 | 506.1 |
|  | **2** |  | 32 | 15.4 | | 499.2 ± 46.6 | | 2.93 ± 0.38 | 0.0 | na | 0.00 | 0.0 | 15.3 | 499.2 |
|  | **3** |  | 4 | 15.4 | | 108.1 ± 15.4 | | 1.51 ± 0.13 | 650.7 ± 83.5 | 6.15 ± 1.63 | 0.00 | 0.0 | 1.6 | 108.1 |
| **3** | **6** | Bahamas | 42 | 15.0 | | 265.8 ± 22.7 | | 9.82 ± 0.97 | 68.5 ± 6.5 | 9.68 ± 1.06 | 0.4 ± 0.3 | 8.3 ± 4.1 | 38.9 | 343.0 |
|  | **7** |  | 42 | 15.0 | | 772.3 ± 63.1 | | 9.87 ± 0.89 | 66.3 ± 9.2 | 15.89 ± 1.19 | 2.1 ± 1.5 | 12.4 ± 4.4 | 88.2 | 853.1 |
| **4** | **8** | Cuba | 60 | 16.0 | | 667.4 ± 48.9 | | 11.00 ± 0.69 | 0.4 ± 0.4 | 12.07 ± 1.00 | 0.2 ± 0.1 | 0.7 ± 0.3 | 65.5 | 668.6 |
|  | **9** |  | 60 | 16.0 | | 595.8 ± 35.5 | | 12.13 ± 0.75 | 0.0 | na | 1.9 ± 1.7 | 10.1 ± 6.5 | 77.8 | 607.9 |
| **5** | **10** | Mexico | 92 | 20.0 | | 683.1 ± 16.1 | | 10.23 ± 0.23 | 199.3 ± 10.6 | 11.04 ± 0.46 | 23.6 ± 1.8 | 17.6 ± 4.0 | 133.2 | 923.6 |
|  | **11** |  | 92 | 20.0 | | 524.1 ± 15.9 | | 7.53 ± 0.25 | 115.9 ± 7.6 | 10.51 ± 0.33 | 27.1± 1.8 | 39.0 ± 5.1 | 117.0 | 706.0 |
|  | **12** |  | 91 | 20.0 | | 667.3± 19.2 | | 5.68 ± 0.20 | 51.7 ± 3.7 | 8.59 ± 0.44 | 19.2 ± 1.2 | 0.4 ± 0.2 | 61.5 | 738.6 |
|  | **13** |  | 77 | 20.0 | | 725.6 ± 17.6 | | 7.80 ± 0.22 | 310.7 ± 12.2 | 13.36 ± 0.40 | 36.6 ± 2.5 | 192.1 ± 13.4 | 325.5 | 1264.9 |
| **6*** | **14** | Mexico | 16 | 20.0 | | 1223.0 ± 177.7 | | 15.50 ± 6.50 | 0.0 | na | 0.00 | 1.1 ± 1.0 | 217.6 | 1224.1 |
| **7** | **15** | Cayman Isl. | 8 | 15.2 | | 1014.6 ± 113.6 | | 8.32 ± 1.83 | 0.0 | na | 5.1 ± 3.4 | 0.0 | 83.1 | 1019.7 |
|  | **16** |  | 6 | 15.2 | | 914.9 ± 99.2 | | 5.95 ± 0.71 | 0.0 | na | 3.7 ± 3.7 | 0.0 | 57.2 | 918.7 |
| **8** | **17** | Jamaica | 36 | 16.0 | | 852.1 ± 44.9 | | 13.12 ± 1.04 | 0.0 | na | 6.8 ± 1.5 | 0.2 ± 0.2 | 114.3 | 859.1 |
|  | **18** |  | 24 | 16.0 | | 1121.6 ± 76.3 | | 11.35 ± 1.13 | 0.0 | na | 11.1 ± 4.4 | 0.0 | 137.9 | 1132.7 |
| **9** | **19** | Dominican R. | 12 | 15.2 | | 629.5± 97.2 | | 9.93 ± 2.40 | 0.0 | na | 40.7 ± 10.1 | 59.3 ± 29.2 | 155.5 | 729.4 |
|  | **20** |  | 4 | 15.2 | | 334.1 ± 61.8 | | 16.25 ± 7.47 | 0.0 | na | 52.4 ± 30.6 | 104.7 ± 100.4 | 211.9 | 491.2 |
| **10** | **21** | Puerto Rico | 48 | 15.2 | | 844.2 ± 45.0 | | 15.87 ± 1.20 | 0.0 | na | 38. 8 ± 7.9 | 18.7 ± 5.4 | 184.8 | 901.9 |
|  | **22** |  | 52 | 15.2 | | 762.7 ± 35.0 | | 16.97 ± 0.98 | 0.0 | na | 3.7 ± 3.7 | 18.9 ± 15.1 | 153.8 | 785.3 |
| **11** | **23** | Belize | 4 | 15.2 | | 1715.3 ± 93.8 | | 8.05 ± 0.49 | 128.9 ± 54.8 | 15.44 ± 2.56 | 25.7 ± 10.3 | 0.0 | 180.5 | 1869.9 |
|  | **24** |  | 4 | 15.2 | | 906.8 ± 212.9 | | 6.69 ± 0.19 | 33.9 ± 20.2 | 18.42 ± 6.42 | 11.2 ± 2.1 | 0.0 | 77.3 | 951.8 |
| **12** | **25** | Belize | 92 | 15.2 | | 1960.2 ± 59.4 | | 4.29 ± 0.13 | 57.9 ± 6.9 | 17.80 ± 2.23 | 42.7 ± 5.6 | 2.8 ± 1.1 | 136.9 | 2063.7 |
|  | **26** |  | 72 | 15.2 | | 1708.3 ± 49.4 | | 3.70 ± 0.13 | 80.1 ± 16.4 | 18.43 ± 0.85 | 6.0 ± 1.2 | 1.4 ± 1.3 | 83.0 | 1795.8 |
| **13** | **27** | Colombia | 20 | 10.7 | | 673.6 ± 105.1 | | 10.12 ± 1.77 | 49.7 ± 11.6 | 14.33 ± 2.61 | 3.2 ± 1.7 | 4.4 ± 2.5 | 67.3 | 730.9 |
|  | **28** |  | 16 | 10.7 | | 525.8 ± 70.9 | | 13.79 ± 1.71 | 74.8 ± 22.3 | 9.49 ± 2.02 | 13.5 ± 12.4 | 14.8 ± 5.6 | 100.5 | 629.0 |
|  | **29** | Colombia | 24 | 10.7 | | 260.2 ± 31.7 | | 12.91 ± 1.70 | 222.2 ± 24.8 | 21.19 ± 2.27 | 2.3 ± 0.9 | 13.7 ± 8.7 | 89.0 | 498.4 |
|  | **30** |  | 24 | 10.7 | | 317.3 ± 31.6 | | 10.56 ± 1.55 | 152.8 ± 27.3 | 19.39 ± 1.69 | 3.2 ± 1.6 | 7.7 ± 5.7 | 70.5 | 481.0 |
|  | **31** | Colombia | 24 | 10.7 | | 629.3 ± 167.8 | | 12.35 ± 1.53 | 160.2 ± 21.0 | 21.59 ± 2.70 | 15.5 ± 4.3 | 4.0 ± 1.9 | 106.9 | 809.0 |
|  | **32** |  | 20 | 10.7 | | 563.0 ± 146.4 | | 11.94 ± 1.61 | 87.1 ± 14.0 | 20.88 ± 2.84 | 12.5 ± 5.7 | 7.7 ± 5.4 | 84.7 | 670.4 |
| **14** | **33** | Barbados | 35 | | 16.0 | 1117.5 ± 167.0 | | 6.16 ± 0.84 | 1066.0 ± 53.5 | 13.22 ± 0.72 | 6.9 ± 2.4 | 2.5 ± 1.8 | 211.1 | 2193.0 |
|  | **34** |  | 32 | | 16.0 | 1193.2 ± 118.4 | | 7.14 ± 0.71 | 807.1 ± 70.9 | 13.38 ± 0.98 | 6.3± 2.3 | 3.4 ± 1.9 | 187.7 | 2010.0 |
| **15** | **37** | Colombia | 32 | | 10.7 | 514.1 ± 42.5 | | 12.02 ± 0.92 | 116.2 ± 11.8 | 21.04 ± 1.60 | 45.7 ± 6.4 | 3.7 ± 2.2 | 131.5 | 679.8 |
|  | **38** |  | 32 | | 10.7 | 624.9 ± 40.8 | | 11.84 ± 0.98 | 23.6 ± 11.5 | 19.61 ± 3.92 | 40.9 ± 6.6 | 1.1 ± 0.6 | 116.6 | 690.5 |
| **16** | **39** | Curaçao | 12 | | 16.0 | 310.5 ± 49.5 | | 7.36 ± 0.93 | 0.0 | na | 20.0 ± 4.2 | 7.0 ± 5.9 | 49.7 | 337.4 |
| **17** | **41** | Colombia | 48 | | 15.0 | 889.0 ± 49.1 | | 7.57 ± 0.54 | 0.0 | na | 19.2 ± 4.6 | 0.2 ± 0.2 | 80.5 | 908.3 |
|  | **42** |  | 48 | | 15.0 | 933.0 ± 65.7 | | 7.10 ± 0.49 | 0.0 | na | 20.1 ± 4.9 | 0.5 ± 0.2 | 83.6 | 953.6 |
| **18** | **43** | Tobago | 96 | | 14.9 | 361.6 ± 17.9 | | 21.01 ± 0.66 | 0.0 | na | 0.5 ± 0.3 | 0.4 ± 0.3 | 77.0 | 362.5 |
|  | **44** |  | 70 | | 14.9 | 285.4 ± 15.7 | | 22.14 ± 0.53 | 0.0 | na | 0.0 | 0.0 | 62.6 | 285.4 |
|  | **45** |  | 4 | | nd | 520.9 ± 46.7 | | 16.50 ± 2.71 | 0.0 | na | 0.0 | 0.0 | 82.7 | 520.9 |
| **20** | **47** | Venezuela | 54 | | 15.5 | 891.1 ± 47.5 | | 16.47 ± 0.77 | 0.0 | na | 28.1 ± 8.9 | 0.0 | 169.9 | 919.2 |
|  | **48** |  | 40 | | 15.5 | 926.0 ± 58.7 | | 17.62 ± 2.70 | 0.0 | na | 38.8 ± 11.3 | 9.3 ± 5.3 | 189.2 | 974.2 |
| **21** | **49** | Costa Rica | 16 | | 15.5 | 815.7 ± 74.9 | | 6.98 ± 0.41 | 0.0 | na | 0.0 | 3.6 ± 3.0 | 59.8 | 819.3 |
|  | **50** |  | 12 | | 15.5 | 885.2 ± 95.1 | | 9.28 ± 0.85 | 15.3 ± 11.3 | 22.77 ± 0.14 | 18.4 ± 13.6 | 0.0 | 104.5 | 918.9 |
| **22** | **51** | Panama | 124 | | 15.2 | 415.5± 10.1 | | 16.40 ± 0.49 | 0.0 | na | 0.6 ± 0.2 | 0.1 ± 0.1 | 69.2 | 416.3 |
|  | **52** |  | 120 | | 15.2 | 365.9 ± 14.5 | | 23.20 ± 0.88 | 0.0 | na | 0.0 | 0.0 | 79.7 | 365.9 |
