# Supplementary material for: Caribbean-Wide, Long-Term Study of Seagrass Beds Reveals Local Variations, Shifts in Community Structure and Occasional Collapse
Source: PLoS One. 2014 Mar 3;9(3):e90600. doi: 10.1371/journal.pone.0090600 (PMC4036797; doi:10.1371/journal.pone.0090600)
Supplement: Table S4 — Intra-annual variability in Thalassia testudinum leaf productivity. Results of One-sample t-test for significant differences of ΔP (deviations from general mean leaf productivity) during High- and Low-growth season at different latitudes. H0: Average ΔP = 0, α = 0.05. (DOCX) [file pone.0090600.s006.docx]

**Table S4**

**Intra-annual variability in *Thalassia testudinum* leaf productivity.**

Results of One-sample t-test for significant differences of ΔP (deviations from general mean leaf productivity) during High- and Low-growth season at different latitudes. H0: Average ΔP =0, α=0.05.

|  |  | **High-Growth Season** | |  | **Low-growth Season** | |  |
| --- | --- | --- | --- | --- | --- | --- | --- |
| **Latitude** | **Location** | **t** | **df** | **p** | **t** | **df** | **p** |
| **High-growth season** |  |  |  |  |  |  |  |
| 9°21'  9°44'  10°52'  11°18'  11°19' | 22  21  20  18  17 | 1.265  0.004  0.124  0.988  1.574 | 31  20  11  42  6 | ns  ns  ns  ns  ns | -1.415  -0.712  0.224  -1.168  -1.823 | 27  10  8  21  16 | ns  ns  ns  ns  ns |
| 13°04' | 14 | 0.659 | 8 | ns | -0.712 | 11 | ns |
| 16°48' | 12 | 2.573 | 23 | 0.017 | -3.642 | 23 | 0.001 |
| 20°53' | 5 | 4.667 | 57 | <0.001 | -7.986 | 54 | <0.001 |
| 22°31' | 4 | 2.001 | 16 | ns | -2.597 | 15 | 0.020 |
| 24°48' | 2 | 4.697 | 26 | <0.001 | -7.614 | 28 | <0.001 |
| 32°24' | 1 | 4.262 | 17 | 0.001 | -5.943 | 7 | 0.001 |
