# Supplementary material for: Caribbean-Wide, Long-Term Study of Seagrass Beds Reveals Local Variations, Shifts in Community Structure and Occasional Collapse
Source: PLoS One. 2014 Mar 3;9(3):e90600. doi: 10.1371/journal.pone.0090600 (PMC4036797; doi:10.1371/journal.pone.0090600)
Supplement: Table S5 — Correlations between temperature, light and Thalassia testudinum leaf growth. Correlations between mean monthly SST (Sea Surface temperature, °C), H daylight (Hours of daylight) and shoot growth rates of Thalassia testudinum at USA-Florida Keys (Site 2, 1996–2003), Mexico-Puerto Morelos (Site 5, 1990–1991: from Van Tussenbroek BI [1995] Thalassia testudinum leaf dynamics in a Mexican Caribbean reef lagoon. Mar Biol 122: 33–40) and Tobago-Bon Accord Lagoon (Site 18, 1997–2007), N number of months, ns: not significant. Hours daylight were obtained from http://astro.unl.edu/classaction/animations/coordsmotion/daylighthoursexplorer.html. Mean monthly SST were from: NOAA Coral Reef Watch, Coral Bleaching Virtual Stations (http://www.osdpd.noaa.gov/ml/ocean/cb/virtual_stations.html )-Sombrero Reef, Florida (Site 2), Rodríguez-Martínez RE, Ruíz-Rentería F, Van Tussenbroek BI, Barba-Santos G, Escalante-Mancera E et al. [2010] State and environmental tendencies of the Puerto Morelos CARICOMP site, Mexico. Rev Biol Trop 58: 23–43 (Site 5), and R. Juman, J. Gomez [unpublished data] (Site 18). (DOCX) [file pone.0090600.s007.docx]

**Table S5.**

**Correlations between temperature, light and *Thalassia testudinum* leaf growth.**

Correlations between mean monthly SST (Sea Surface temperature, ^o^C), H daylight (Hours of daylight) and shoot growth rates of *Thalassia testudinum* at USA-Florida Keys (Site 2, 1996-2003), Mexico-Puerto Morelos (Site 5, 1990-1991: from Van Tussenbroek BI [1995] *Thalassia testudinum* leaf dynamics in a Mexican Caribbean reef lagoon. Mar Biol 122: 33-40) and Tobago-Bon Accord Lagoon (Site 18, 1997-2007), N number of months, ns: not significant. Hours daylight were obtained from [http://astro.unl.edu/ classaction/ animations/ coordsmotion/ daylighthoursexplorer.html](http://astro.unl.edu/%20classaction/%20animations/%20coordsmotion/%20daylighthoursexplorer.html). Mean monthly SST were from: NOAA Coral Reef Watch, Coral Bleaching Virtual Stations (http://www.osdpd.noaa.gov/ml/ocean/cb/virtual_stations.html)-Sombrero Reef, Florida (Site 2), Rodríguez-Martínez RE, Ruíz-Rentería F, Van Tussenbroek BI, Barba-Santos G, Escalante-Mancera E et al. [2010] State and environmental tendencies of the Puerto Morelos CARICOMP site, Mexico. Rev Biol Trop 58: 23-43 (Site 5), and R. Juman, J. Gomez [unpublished data] (Site 18).

|  | **Sta-** |  | **Lat** | **SST** | | **H daylight** | |
| --- | --- | --- | --- | --- | --- | --- | --- |
| **Site** | **tion** | **N** | **(^o^N)** | **r** | **P** | **r** | **p** |
| **2** | **4** | 8 | 24^o^24´ | 0.79 | 0.021 | 0.90 | 0.002 |
|  | **5** | 8 |  | 0.77 | 0.004 | 0.88 | 0.004 |
| **5** | **10** | 12 | 20^o^54´ | 0.48 | ns | 0.81 | 0.015 |
|  | **12** | 12 |  | 0.42 | ns | 0.82 | 0.008 |
|  | **13** | 12 |  | 0.30 | ns | 0.80 | 0.020 |
| **18** | **44** | 7 | 11^o^18´ | -0.16 | ns | 0.41 | ns |
|  | **45** | 7 |  | 0.18 | ns | 0.08 | ns |
