# Supplementary material for: Caribbean-Wide, Long-Term Study of Seagrass Beds Reveals Local Variations, Shifts in Community Structure and Occasional Collapse
Source: PLoS One. 2014 Mar 3;9(3):e90600. doi: 10.1371/journal.pone.0090600 (PMC4036797; doi:10.1371/journal.pone.0090600)
Supplement: Table S6 — Regression lines of trends. Results for the linear regressions of selected parameters vs year to indicate possible trends Regressions were computed for those stations and parameters when the sampling covered at least 5 y with at least six sampling events and at least 50% of the samples had values >0. *: not determined, ns: not significant, negative t indicated a negative slope. The significance level (α) is 0.05, but a Bonferroni correction is applied to this level of significance, because the parameters are derived from the same cores (Total above-ground biomass. Relative abundance of faster-growing seagrass, Relative abundance of faster growing fleshy algae, % Above-ground/total biomass for Thalassia testudinum) or quadrats (Productivity, Foliar shoot density of T. testudinum). The results of the regressions of foliar weight per shoot of T. testudinum is also given, to facilitate interpretations of the results, although this was not a parameter for potential degradation of the coastal environment. (DOCX) [file pone.0090600.s008.docx]

**Table S6.**

**Regression lines of trends.**

Results for the linear regressions of selected parameters *vs* year to indicate possible trends Regressions were computed for those stations and parameters when the sampling covered at least 5 y with at least six sampling events and at least 50% of the samples had values >0. *: not determined, ns: not significant, negative t indicated a negative slope. The significance level (α) is 0.05, but a Bonferroni correction is applied to this level of significance, because the parameters are derived from the same cores (Total above-ground biomass. Relative abundance of faster-growing seagrass, Relative abundance of faster growing fleshy algae, % Above-ground / total biomass for *Thalassia testudinum*) or quadrats (Productivity, Foliar shoot density of *T. testudinum*). The results of the regressions of foliar weight per shoot of *T. testudinum* is also given, to facilitate interpretations of the results, although this was not a parameter for potential degradation of the coastal environment.

| **Site** | **Sta-tion** | **Country/**  **Territory** |  | | **Community** | | | **Relative abundance of faster –growing plants** | | | | | | ***Thalassia testudinum*** | | | |
| --- | --- | --- | --- | --- | --- | --- | --- | --- | --- | --- | --- | --- | --- | --- | --- | --- | --- |
|  |  |  |  | | **Total above-ground biomass** | | | **Other seagrass** | | | **Fleshy Algae^1^** | | | **% Above-ground / total biomass** | | | |
|  |  |  | **df** | **α** | **slope** | **t** | **p** | **slope** | **t** | **p** | **slope** | **t** | **p** | **slope** | **t** | | **p** |
| **1** | **1** | Bermuda | 36 | 0.025 | -93.72 | -7.51 | 0.001 | * | * | * | * | * | * | -0.68 | | -6.13 | 0.001 |
|  | **2** |  | 31 | 0.025 | -63.84 | -3.45 | 0.002 | * | * | * | * | * | * | -0.53 | | -3.49 | 0.002 |
| **2** | **4** | USA^2^ | * | * | ns | -1.08 | 0.94 | * | * | * | * | * | * | * | | * | * |
|  | **5** |  | * | * | 5.31 | 4.77 | 0.001 | * | * | * | * | * | * | * | | * | * |
| **3** | **6** | Bahamas | 41 | 0.017 | ns | 1.82 | 0.076 | ns | 2.34 | 0.024 | * | * | * | ns | | -1.50 | 0.141 |
|  | **7** |  | 41 | 0.017 | ns | -0.52 | 0.605 | -0.93 | -2.72 | 0.010 | ns | -1.26 | 0.216 | ns | | -2.11 | 0.041 |
| **4** | **8** | Cuba | 59 | 0.025 | -5.27 | -2.60 | 0.012 | * | * | * | * | * | * | 0.78 | | 3.26 | 0.002 |
|  | **9** |  | 59 | 0.025 | -7.98 | -2.28 | 0.025 | * | * | * | * | * | * | 0.96 | | 2.94 | 0.005 |
| **5** | **10** | Mexico | 91 | 0.013 | ns | 0.88 | 0.380 | 1.30 | 5.86 | 0.001 | 0.01 | 4.12 | 0.001 | 0.12 | | 2.57 | 0.012 |
|  | **11** |  | 91 | 0.013 | 4.04 | 3.57 | 0.001 | ns | 0.27 | 0.791 | 0.02 | 6.45 | 0.001 | 0.21 | | 3.90 | 0.001 |
|  | **12** |  | 90 | 0.017 | ns | 1.39 | 0.169 | 0.56 | 4.26 | 0.001 | * | * | * | 0.21 | | 5.54 | 0.001 |
|  | **13** |  | 76 | 0.013 | 8.68 | 2.58 | 0.010 | ns | 0.82 | 0.413 | ns | 1.99 | 0.072 | 0.24 | | 4.12 | 0.001 |
| **7** | **15** | Cayman Isl. | na | na | * | * | * | * | * | * | * | * | * | * | | * | * |
| **8** | **17** | Jamaica | 35 | 0.025 | 9.70 | 2.38 | 0.024 | * | * | * | * | * | * | 1.11 | | 2.02 | 0.050 |
| **10** | **21** | Puerto | 47 | 0.017 | 19.08 | 5.67 | 0.001 | * | * | * | 0.75 | 2.79 | 0.002 | 0.88 | | 3.38 | 0.001 |
|  | **22** | Rico | 51 | 0.017 | ns | 1.86 | 0.069 | * | * | * | ns | 0.22 | 0.823 | 0.90 | | 4.52 | 0.001 |
| **12** | **25** | Belize | 91 | 0.013 | ns | -0.84 | 0.403 | 0.57 | 4.82 | 0.001 | ns | 1.28 | 0.226 | 0.10 | | 6.16 | 0.001 |
|  | **26** |  | 71 | 0.013 | ns | -1.22 | 0.226 | ns | 2.09 | 0.042 | ns | -0.12 | 0.904 | 0.07 | | 2.99 | 0.004 |
| **13** | **29** | Old | 23 | 0.017 | ns | 1.95 | 0.064 | ns | 0.68 | 0.505 | * | * | * | ns | | -1.04 | 0.310 |
|  | **30** | Providence | 23 | 0.017 | ns | 0.94 | 0.358 | -6.46 | -3.65 | 0.001 | * | * | * | ns | | 0.40 | 0.694 |
|  | **31** |  | 23 | 0.017 | ns | 0.42 | 0.679 | ns | 0.41 | 0.684 | * | * | * | ns | | 0.26 | 0.800 |
| **14** | **33** | Barbados^3.^ | 34 | 0.017 | ns | -0.03 | 0.980 | 3.55 | 3.41 | 0.002 | * | * | * | 0.74 | | 5.43 | 0.001 |
|  | **34** |  | 31 | 0.013 | ns | -1.31 | 0.200 | 0.84 | 2.75 | 0.010 | ns | 0.70 | 0.521 | 1.13 | | 5.32 | 0.001 |
| **15** | **37** | San Andres | 31 | 0.017 | ns | -0.79 | 0.438 | ns | -1.92 | 0.064 | * | * | * | ns | | -0.46 | 0.650 |
|  | **38** |  | 31 | 0.017 | ns | -0.65 | 0.519 | ns | 0.94 | 0.357 | * | * | * | ns | | 1.17 | 0.253 |
| **17** | **41** | Colombia | 47 | 0.025 | ns | -1.50 | 0.141 | * | * | * | * | * | * | ns | | 2.26 | 0.028 |
|  | **42** |  | 47 | 0.025 | ns | -1.16 | 0.251 | * | * | * | * | * | * | ns | | 2.23 | 0.030 |
| **18** | **43** | Tobago | 95 | 0.025 | ns | 0.89 | 0.377 | * | * | * | * | * | * | ns | | 0.82 | 0.417 |
|  | **44** |  | 69 | 0.025 | ns | -1.61 | 0.113 | * | * | * | * | * | * | ns | | 0.70 | 0.486 |
| **20** | **47** | Venezuela | 53 | 0.025 | 12.56 | 5.31 | 0.001 | * | * | * | * | * | * | 0.37 | | 2.31 | 0.025 |
|  | **48** |  | 39 | 0.025 | 25.23 | 4.20 | 0.001 | * | * | * | * | * | * | 1.22 | | 3.81 | 0.001 |
| **21** | **49** | Costa Rica | 15 | 0.025 | -8.00 | -2.53 | 0.023 | * | * | * | * | * | * | ns | | 0.61 | 0.550 |
|  | **50** |  | * | * | * | * | * | * | * | * | * | * | * | * | | * | * |
| **22** | **51** | Panama | 123 | 0.050 | 3.10 | 5.28 | 0.001 | * | * | * | * | * | * | * | | * | * |
|  | **52** |  | 119 | 0.050 | 5.61 | 6.75 | 0.001 | * | * | * | * | * | * | * | | * | * |

**Table S6 (continued)**

| **Site** | **Sta-tion** | **Country/Territory** |  | | ***Thalassia testudinum*** | | | |  | | | | | | |  |
| --- | --- | --- | --- | --- | --- | --- | --- | --- | --- | --- | --- | --- | --- | --- | --- | --- |
|  |  |  |  | | **Productivity** | | |  | **Foliar shoot density** | | | | **Dry weight per Shoot** | | |  |
|  |  |  | **df** | **Α** | **slope** | **t** | **p** | **df^4.^** | | **slope** | **t** | **p** | **slope** | **t** | **p** | |
| **1** | **1** | Bermuda | 87 | 0.025 | -0.11 | -5.67 | 0.001 | 75 | | 80.3 | 6.57 | 0.001 | -7.16 | -7.79 | 0.001 | |
|  | **2** |  | 74 | 0.025 | -0.24 | -9.76 | 0.001 | 63 | | -58.3 | -3.82 | 0.001 | -9.56 | -7.32 | 0.001 | |
| **2** | **4** | USA^2^ | 167 | 0.025 | ns | 0.64 | 0.525 | 167 | | ns | 1.61 | 0.109 | ns | -1.09 | 0.050 | |
|  | **5** |  | 164 | 0.025 | 0.05 | 2.76 | 0.022 | 164 | | 19.2 | 3.61 | 0.001 | 6.96 | 3.07 | 0.002 | |
| **3** | **6** | Bahamas | * | na | * | * | * | * | | * | * | * | * | * | * | |
|  | **7** |  | * | na | * | * | * | * | | * | * | * | * | * | * | |
| **4** | **8** | Cuba | 95 | 0.025 | ns | -1.88 | 0.063 | 82 | | -58.9 | -5.72 | 0.001 | 9.42 | 3.28 | 0.002 | |
|  | **9** |  | 101 | 0.025 | ns | 1.46 | 0.148 | 89 | | -36.3 | -3.79 | 0.001 | 10.13 | 4.11 | 0.001 | |
| **5** | **10** | Mexico | 178 | 0.025 | 0.04 | 3.19 | 0.002 | 178 | | 11.3 | 4.31 | 0.001 | 2.81 | 3.58 | 0.001 | |
|  | **11** |  | 173 | 0.025 | ns | 1.60 | 0.112 | 173 | | 8.9 | 2.92 | 0.004 | ns | -0.22 | 0.827 | |
|  | **12** |  | 163 | 0.025 | 0.02 | 2.79 | 0.006 | 163 | | ns | 1.16 | 0.249 | 0.89 | 3.34 | 0.001 | |
|  | **13** |  | 139 | 0.025 | ns | 0.99 | 0.324 | 139 | | ns | -1.54 | 0.126 | 3.15 | 3.87 | 0.001 | |
| **7** | **15** | Cayman Isl. | 35 | 0.025 | 0.43 | 5.30 | 0.001 | 35 | | -62.2 | -4.04 | 0.001 | 9.11 | 4.77 | 0.001 | |
| **8** | **17** | Jamaica | 34 | 0.050 | 0.26 | 2.17 | 0.037 | * | | * | * | * | * | * | * | |
| **10** | **21** | Puerto | 47 | 0.025 | ns | 1.46 | 0.151 | 47 | | ns | 0.74 | 0.463 | ns | 0.93 | 0.358 | |
|  | **22** | Rico | 47 | 0.025 | ns | 0.64 | 0.524 | 47 | | ns | 1.84 | 0.072 | ns | 1.38 | 0.173 | |
| **12** | **25** | Belize | 167 | 0.025 | 0.06 | 4.505 | 0.001 | 155 | | -14.7 | -5.48 | 0.001 | 5.07 | 7.19 | 0.001 | |
|  | **26** |  | 115 | 0.025 | ns | 1.944 | 0.054 | 115 | | 18.0 | 5.18 | 0.001 | -1.27 | -2.37 | 0.020 | |
| **13** | **29** | Old | 34 | 0.025 | ns | -0.49 | 0.628 | ns | | 34 | -0.43 | 0.668 | ns | 0.15 | 0.880 | |
|  | **30** | Providence | 34 | 0.025 | ns | -1.89 | 0.068 | ns | | 34 | -0.90 | 0.377 | ns | 0.75 | 0.451 | |
|  | **31** |  | 29 | 0.025 | -0.31 | -3.131 | 0.004 | ns | | 29 | 1.26 | 0.217 | -24.9 | -2.60 | 0.015 | |
| **14** | **33** | Barbados^3.^ | 63 | 0.025 | ns | -1.54 | 0.128 | 27 | | -59.6 | -3.24 | 0.003 | 25.33 | 4.32 | 0.001 | |
|  | **34** |  | 55 | 0.025 | ns | -1.27 | 0.210 | 23 | | * | * | * | * | * | * | |
| **15** | **37** | Colombia | 40 | 0.025 | ns | -1.25 | 0.230 | 40 | | 55.5 | 3.13 | 0.003 | -22.6 | -2.93 | 0.006 | |
|  | **38** |  | 37 | 0.025 | ns | -1.01 | 0.320 | 37 | | ns | 1.19 | 0.243 | ns | -2.14 | 0.039 | |
| **17** | **41** | Colombia | 71 | 0.025 | ns | -1.49 | 0.141 | 71 | | ns | 0.99 | 0.326 | -5.11 | -5.12 | 0.001 | |
|  | **42** |  | 71 | 0.025 | ns | -1.06 | 0.293 | 71 | | ns | 0.05 | 0.961 | ns | -1.34 | 0.186 | |
| **18** | **43** | Tobago | 201 | 0.025 | ns | 0.26 | 0.799 | 173 | | ns | -0.04 | 0.971 | ns | 0.59 | 0.558 | |
|  | **44** |  | 181 | 0.025 | -0.10 | -2.62 | 0.009 | 168 | | ns | 1.41 | 0.160 | -20.33 | -3.90 | 0.001 | |
| **20** | **47** | Venezuela | 59 | 0.050 | 0.24 | 6.98 | 0.001 | * | | * | * | * | * | * | * | |
|  | **48** |  | 41 | 0.050 | 0.16 | 2.65 | 0.011 | * | | * | * | * | * | * | * | |
| **21** | **49** | Costa Rica | 104 | 0.025 | -0.23 | -6.59 | 0.001 | 104 | | -42.9 | -2.96 | 0.004 | -3.89 | -4.44 | 0.001 | |
|  | **50** |  | 73 | 0.025 | -0.39 | -2.85 | 0.006 | 73 | | ns | -0.68 | 0.496 | ns | -1.34 | 0.186 | |
| **22** | **51** | Panama | 161 | 0.025 | ns | -0.49 | 0.622 | 161 | | ns | -0.36 | 0.717 | ns | 0.27 | 0.785 | |
|  | **52** |  | 164 | 0.025 | ns | 1.42 | 0.156 | 164 | | 32.88 | 6.97 | 0.001 | -1.94 | -3.71 | 0.001 | |

1. Distribution of the fleshy algae is irregular and samples were grouped per sampling time, 2. Above-ground biomass at site 2 was determined from leaf dry weight of the quadrat samples (α=0.025), 3. Trend for above-ground relative to total biomass was determined for *Syringodium filiforme*, because *Thalassia testudinum* disappeared in later years at this site, 4. Df for shoot density may be lower than the value for productivity because this parameter was included later in the protocol.
